# Unveiling attenuation structures in the northern Taiwan volcanic zone

**DOI:** 10.1038/s41598-024-55397-8

**Published:** 2024-02-27

**Authors:** Y.-P. Lin, J. Y.-T. Ko, B.-S. Huang, C.-H. Lin, M.-H. Shih

**Affiliations:** 1 Department of Geosciences, National Taiwan University, Taipei, Taiwan; 2 Institute of Oceanography, National Taiwan University, Taipei, Taiwan; 3 Institute of Earth Sciences, Academia Sinica, Taipei, Taiwan; 4 National Center for Research on Earthquake Engineering, Taipei, Taiwan

**Keywords:** Geophysics, Seismology

## Abstract

This cutting-edge study delves into regional magmatism in northern Taiwan through advanced 3-D *P*- and *S*-wave frequency-dependent attenuation tomography. Positioned at the dynamic convergence boundary between the Philippine Sea Plate and the Eurasian Plate, Taiwan experiences moderate earthquakes and intriguing volcanic activity, with a focus on the Tatun volcano group. Employing the Formosa seismic array for high-resolution results, our research identifies high-attenuation anomalies (low *Q*) beneath the northern Taiwan volcanic zone (NTVZ) and offshore submarine volcanoes, indicative of potential hydrothermal activities and magma reservoirs at varying depths. Additionally, we explore low-attenuation anomalies (high *Q*) in the forearc region of the Ryukyu subduction zone, suggestive of partial saturation linked to serpentinization processes resulting from seawater infiltration or forearc mantle hydration. These findings shed light on the complex geological features and provide essential insights into the crustal properties of northern Taiwan, contributing to a deeper understanding of its magmatic evolution and tectonic processes.

## Introduction

The northern region of Taiwan has experienced significant tectonic alterations as a result of the transition from oblique collision to post-collisional extension. This has led to the formation of complex onshore and offshore volcanic groups^1,2^. The ongoing subduction of the Philippine Sea Plate (PSP) offshore eastern Taiwan has resulted in the creation of the Ryukyu volcanic arc and submarine volcanoes in the Okinawa trough (OT). The northern Taiwan volcanic zone (NTVZ) was formed from offshore to the inland, believed to be a result of the collapse of the mountain belt due to extension^1,3^. The Tatun volcano group (TVG), located near the western boundary of PSP subduction and close to Taipei city, is a significant component of the NTVZ and poses a risk to communities in northern Taiwan, warranting concern^4^.

The TVG was previously considered to be a dormant or extinct volcano, with its most recent active phase occurring between 2.8 and 0.1 million years ago^4,5^. However, recent dating of volcanic ash in the northern Taipei basin and volcanic debris flow in the TVG has indicated younger eruption events from 20,000 to 6000 years ago^6,7^. The elevated ^3^He/^4^He ratio in the volcanic gases from the TVG hot springs provides evidence of an active magma reservoir beneath the TVG^8,9^. Seismic studies point to the presence of a magma chamber located 30 km beneath the TVG, as evidenced by *S*-wave shadows and *P*-wave travel-time delays from offshore subduction earthquakes^10^. The existence of a magma chamber beneath the TVG was initially challenged in a seismic waveform study^11^. Nevertheless, subsequent confirmation was provided by a *P*-wave tomographic model, pinpointing the magma chamber's location at a depth of 8–20 km beneath the eastern part of the TVG^12^.

Seismic attenuation also provides valuable information about an active plumbing system as the presence of hot magma or melting materials can greatly dissipate seismic energy^13–15^. However, accurately determining seismic attenuation is difficult due to the interplay between source and path effects^16^ and frequency-dependent phenomena arising from the complexities of subsurface structures. Previous spectra-fitting techniques, such as event clustering^16^ and empirical green’s function (EGF) approaches^17–19^, can reduce uncertainty from the source. However, these methods require closely spaced events and may not be suitable for sparsely distributed source areas. Additionally, the limitations of the spectra fitting approaches in accurately capturing frequency-dependent patterns of seismic attenuation stem from the simple seismic response function^20^ used to describe the shape of the spectra. Recently, alternative methods have been developed to overcome the limitations and accurately estimate the frequency-dependent attenuation structures in Southern California^21,22^.

**Figure 1:**
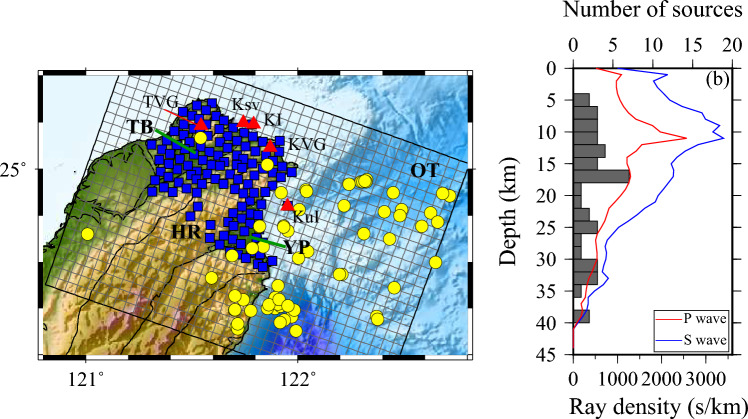
**(a)** Map of the Northern Taiwan study area (tilted box) showing earthquake sources (yellow circles) and Formosa Array (blue squares). Thin lines are the 5-km square grid used here to represent lateral variations in attenuation. Red triangles indicate the volcanoes^72^: TVG Tatun volcano group, KVG Keelung volcano group, KuI Kueishantao Island, KI Keelungyu Island and Ksv Keelung submarine volcano. HR indicates the location of Hsuishan Range. TB is the Taipei basin. YP is the Yilan plain. OT indicates the Okinawa trough**. (b)** Ray density as a function of depth summed over all *P-*wave paths (red curve) and *S*-wave paths (blue curve) ray-traced through the 3D velocity tomography model^31^. Histogram shows the depth distribution of the sources. This figure is generated by GMT 4.5.18 https://www.generic-mapping-tools.org/^73^.

Commencing in 2017, the deployment of the Formosa Array (FMarray) in northern Taiwan sought to better understand the geometry of the magma chamber beneath the Tatun volcanic region. By December 2019, the array was comprised of 146 seismic stations uniformly spaced at 5-km intervals, encompassing both plain and mountainous regions (Fig. 1). This network significantly improved the analysis of elastic and anelastic structures in the area. Additionally, the Formosa Array serves as a vital tool for investigating magma reservoirs and enhancing seismic imaging across northern Taiwan. Each of these stations is equipped with a broadband seismometer and continuously collect seismic data at a sampling rate of 100 Hz, transmitting it in real-time via telephone cables or wireless radio connections to the Institute of Earth Sciences at Academia Sinica and the Taiwan Volcano Observatory at Tatun (TVO) in Taipei, Taiwan^23,24^.

In this study, we employed the method proposed by Lin and Jordan (2023) to invert frequency-dependent 1-D attenuation models for northern Taiwan. The resulting 1-D model was then utilized to initialize a 3-D attenuation inversion process. The data from 10 frequency bands, spanning 1 Hz to 10 Hz, was corrected using the frequency-dependent power law rate *α* obtained from the 1-D results, enabling the estimation of lateral variations at a reference frequency of 5 Hz. The novel model offers fresh understanding of the subsurface configuration of the Tatun volcanic region, revealing the existence of a clearly defined magma reservoir beneath the TVG and a shallow crust magma pathway for the NTVZ in northern Taiwan. In addition to the high attenuation zone underneath volcanic areas, there are noteworthy low attenuation anomalies situated along the mountainous region and southeast offshore of Yilan plain, indicating the presence of low-grade metamorphic rocks and serpentinites, respectively. The study highlights the usefulness of the frequency-dependent attenuation inversion approach in reliably quantifying seismic attenuation and characterizing subsurface structures, particularly in regions where sparsely distributed sources may pose a challenge.

## Data measurements and 1-D attenuation models

In this study, *P* and *S* waveforms were collected from 43 earthquakes with moment magnitudes ranging between 3.0 and 4.5, recorded by 118 stations of the FMarray between 2018 and 2019 (Fig. 1). We selected the first arrivals of direct *P* and *S* waves manually, ensuring that they had a signal-to-noise ratio greater than 3. The study utilized 29,470 *P* phases and 31,120 *S* phases for spectral analysis. The amplitude ${A}_{ij}(f)$ of each *P* or *S* waveform was computed across 10 frequency bands ranging from 1 to 10 Hz, utilizing the approach described by Lin and Jordan^21^. The amplitude spectra were determined by integrating the wavelet transform of the ${i}$th event recorded on the ${j}$th station over a 3-s time window that started 0.7 s before the phase arrival time (Supplementary Fig. 1). Modifying the window lengths has minimal impact on our amplitude measurements and inversion results (Supplementary Figs. 2 and 3). The amplitude measurements were subsequently corrected for source-radiation and geometrical-spreading effects using synthetic spectra ${\widetilde{A}}_{ij}(f)$ computed by a frequency-wavenumber (F-K) simulation^25^. The corrected amplitude data of *P* and *S* waves exhibited evident frequency-dependent patterns (Supplementary Fig. 4a, b), suggesting significant frequency-dependent attenuation in this region. We employed the method to determine attenuation factors, ${Q}_{P}^{-1}$ and ${Q}_{S}^{-1}$, which characterize the high-frequency energy decay of *P* waves and *S* waves as they propagate through subsurface structures. At low frequencies, the attenuation factor *Q* is often assumed to be frequency independent according to Aki and Richards^26^. However, *Q* is theoretically frequency dependent, following a power-law rate above 1 Hz^27,28^ as

1 $$Q\left({\varvec{x}},f\right)={Q}_{0}\left({\varvec{x}}\right){[f/{f}_{0}]}^{\alpha }; \quad  0< \alpha < 1$$

**Figure 2:**
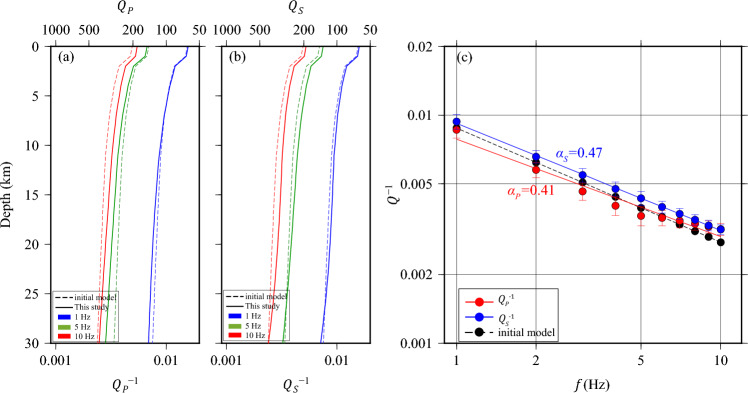
**(a,b)** Depth- and frequency-dependent attenuation models for *P* waves and *S* waves, respectively, sampled at frequencies of 1 Hz (blue), 5 Hz (green), and 10 Hz (red). Dashed lines are the starting model estimated from velocity model^31^; solid lines are the final models from the 1D inversions. **(c)** Red and blue solid circles with 1-sigma error bars are ${Q}_{P}^{-1}$ and ${Q}_{S}^{-1}$ values obtained in this study by separate depth-dependent inversions in 1-Hz bands and depth-averaged from 0 to 30 km, weighted by the ray density of Fig. 1b. Solid red and blue lines are the least-squares fits (${\alpha }_{P}=0.41\pm 0.04, {\alpha }_{S}=0.47\pm 0.04$); dashed black line is the reference model. This figure is generated by GMT 4.5.18 https://www.generic-mapping-tools.org/^73^.

The exponent *α* in Eq. (1) is a measure of the intensity of the frequency dependency, with values ranging between 0 and 1. The value of *Q* at a reference frequency *f*_0_ is represented by *Q*_0_. It is worth noting that estimates of the decay exponent *α* can vary widely in the literature, with reported values ranging from as low as 0.3 to as high as 1^21,29,30^. To better understand the frequency-dependent attenuation behaviors of *P* and *S* waves for northern Taiwan, we inverted the spectral amplitude of *P* and *S* waves independently (Supplementary Fig. 4a, b) and obtain multi-frequency 1-D attenuation models (Fig. 2a). The resulting ${Q}_{P}^{-1}$ and ${Q}_{S}^{-1}$ profiles are compared with the frequency-dependent starting models at frequencies of 1, 5, and 10 Hz (Fig. 2a). The ${Q}_{P}$ models exhibit lower attenuation below 5 Hz but higher attenuation at 10 Hz compared to the initial models, while the ${Q}_{S}$ models consistently display stronger attenuation across all periods of data compared to the initial models. To obtain the frequency dependency, we calculated the average of our optimal 1-D model values across all depths (Fig. 2b). This approach resulted in the best fit of frequency-dependent power-law exponents, with values of *α*_*P*_ = 0.41 ± 0.04 and *α*_*S*_ = 0.47 ± 0.04.

## Lateral variations of attenuation in northern Taiwan

**Figure 3:**
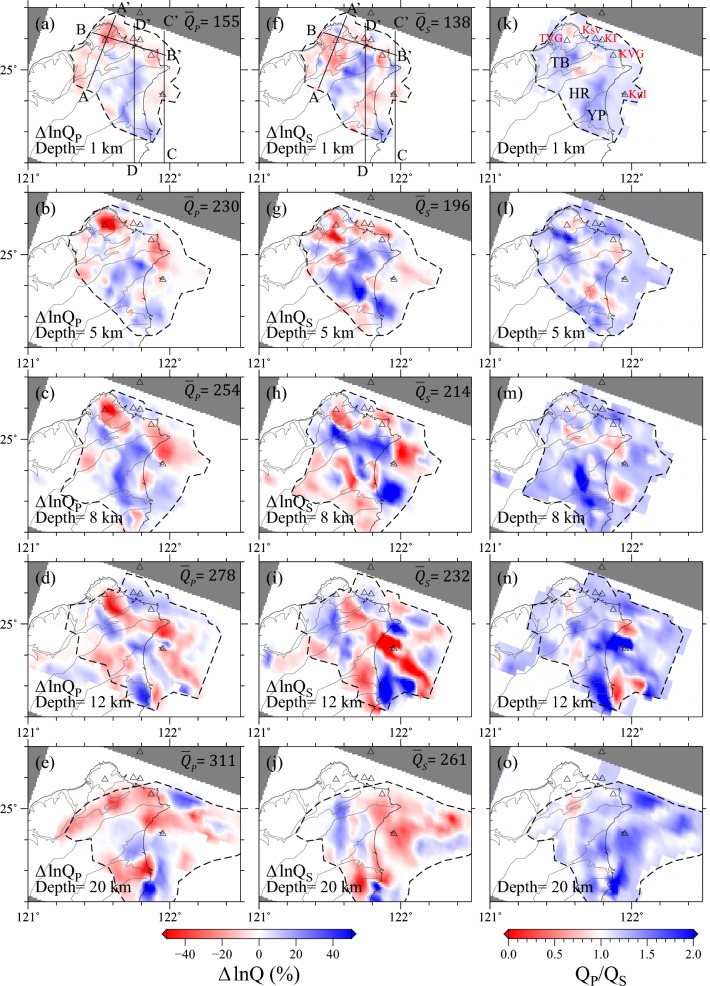
Depth slices at 1 km, 5 km, 8 km, 12 km, and 20 km depths of $\Delta {\text{ln}}{Q}_{P}(\mathbf{x},{f}_{0})$ **(a–e),** $\Delta {\text{ln}}{Q}_{S}(\mathbf{x},{f}_{0})$ **(f–j),** and ${Q}_{P}/{Q}_{S}$ **(k–o)** from the northern Taiwan attenuation tomography model, superposed on the geological zonation map for northern Taiwan. Red colors show stronger attenuation; blue show weaker. Black dashed contours enclose the volume where large-scale features are fairly well resolved by the datasets according to the checkerboard and trial-model inversions in Supplementary Fig. 5. Triangles indicate the locations of volcanoes. Labeled lines in (**a,f**) locate the model cross-sections shown in Fig. 4. The abbreviations for geological zonations and volcanoes are marked in (k), and their full names can be referenced in the caption of Fig. 1. This figure is generated by GMT 4.5.18 https://www.generic-mapping-tools.org/^73^.

**Figure 4:**
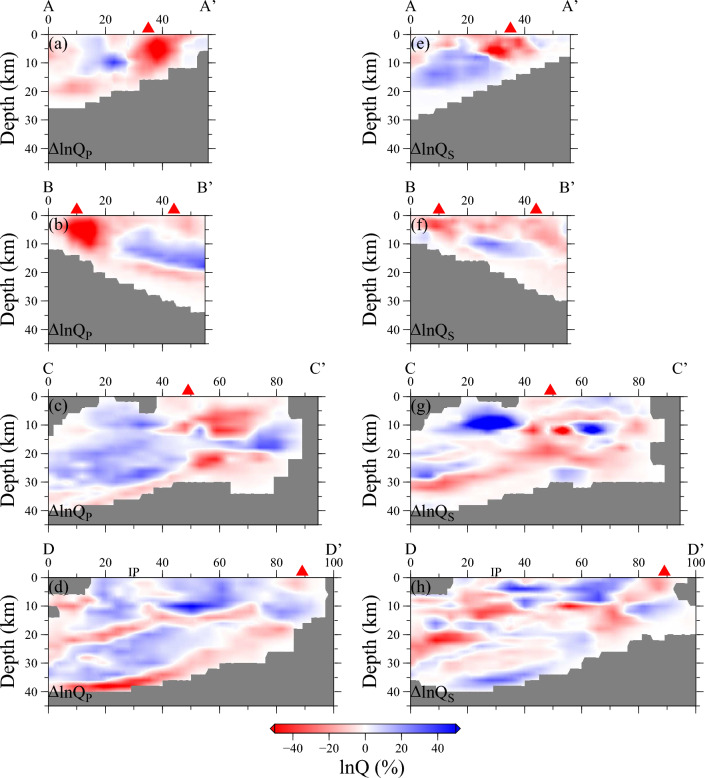
Cross-sections of $\Delta {\text{ln}}{Q}_{P}(\mathbf{x},{f}_{0})$ (left side) and $\Delta {\text{ln}}{Q}_{S}(\mathbf{x},{f}_{0})$ (right side) through the northern Taiwan attenuation tomography model. Cross-sections A-A' to D-D' are located on Fig. 3a, f. Red triangles indicate the locations of volcanoes. This figure is generated by GMT 4.5.18 https://www.generic-mapping-tools.org/^73^.

The 3-D inversion used a model covariance matrix composed of four blocks based on four sets of terms in Eq. (5),${\mathbf{C}}_{s}$, ${\mathbf{C}}_{\Delta {q}_{0}}$, ${\mathbf{C}}_{r0}$, and ${\mathbf{C}}_{\kappa }$, the prior distributions of the source statics ${s}_{i}$, and station statics ${r}_{j0}$ were taken to have large variances and be independent among different elements. Spatial variations of the frequency dependence were ignored, and spectral amplitudes in 1-Hz bands were corrected to a common reference frequency (*f*_0_ = 5 Hz) using the spatially-averaged exponents *α*_*P*_ = 0.41 and *α*_*S*_ = 0.47. The prior means were the updated 5-Hz models of Fig. 2 and the prior covariances were exponential with 50% relative uncertainties and specified lateral and radial smoothing lengths. The study area was parameterized by 35 × 25 × 45 grids (Fig. 1a) with a horizontal spacing of 5 km and layer thickness of 1 km. We computed the Fréchet kernels ${T}_{ij}$(x) in Eq. (5) by 3-D ray tracing through the tomographic velocity model by Huang et al.^31^. In solving the 3-D problem, we separately inverted the *P*-wave dataset (29,470 spectral ratios) for ${Q}_{P}^{-1}\left(\mathbf{x},{f}_{0}\right)$ and the *S*-wave dataset (31,120 spectral ratios) for ${Q}_{S}^{-1}\left(\mathbf{x},{f}_{0}\right)$. The independent inversions achieved the same data variance reductions (60%) and produced well-correlated perturbations in ${Q}_{P}^{-1}$ and ${Q}_{S}^{-1}$ (Figs. 3 and 4 for depth slices and cross-sections, respectively). The checkerboard test is conducted for assessing general resolution power of the inverted attenuation model (Supplementary Fig. 5). The results of the checkerboard tests demonstrated good recovery in most inland and offshore regions of the model.

### Attenuation versus velocity

**Figure 5:**
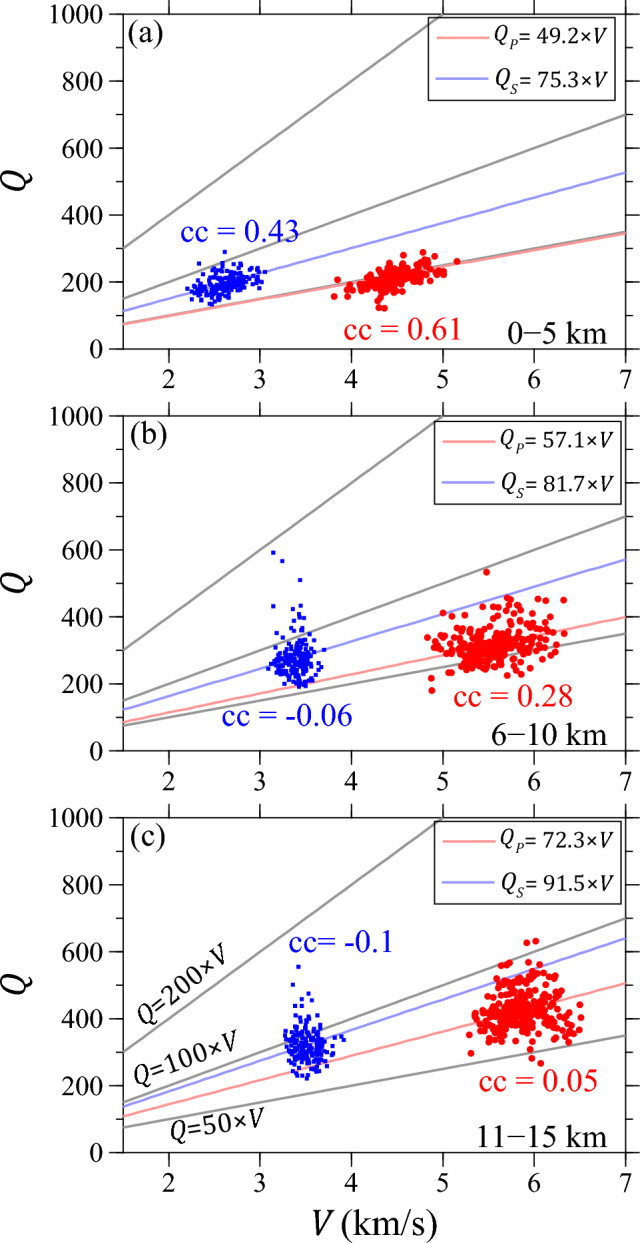
${Q}_{{\text{p}}}$ vs. ${V}_{{\text{p}}}$ (red dots) and ${Q}_{{\text{S}}}$ vs. ${V}_{{\text{S}}}$ (blue squares) for layers 0–5 km (**a**), 6–10 km (**b**) and 11–15 km (**c**) averages in the high-resolution region (Black dashed contours in Fig. 3). Slopes of best-fitting lines (blue and red lines) are labeled in each panel. Correlation coefficients are denoted as “cc” in all panels. This figure is generated by GMT 4.5.18 https://www.generic-mapping-tools.org/^73^.

Previous research on low-frequency ground motions (< 1 Hz) in Southern California typically assumes that the quality factor ${Q}_{S}$ is independent of frequency and directly related to shear velocity ${V}_{S}$ ^32,33^. This correlation is often expressed through the relationship *Q* = *C* × *V*, a formula extensively utilized in strong motion studies to estimate attenuation effects in velocity structures. The constant *C* typically falls within the range of 20 to 150^32,33^ and holds significance as it plays a crucial role in characterizing the intrinsic attenuation properties of the material. A higher *C* value implies a greater overall *Q* value, indicating less attenuation and suggesting that the region is likely composed of colder, harder materials. Conversely, a smaller *C* value indicates a lower overall *Q* value, suggesting that the predominant medium in the region is likely composed of softer materials. In this study, we compared our attenuation model with the latest *P*-wave^12^ and *S*-wave 3-D velocity models^34^ in northern Taiwan (Fig. 5) for three layers at depths of 0–5 km, 6–10 km, and 11–15 km.

As depth increases, the *C* value progressively becomes larger, indicating a more rapid increase in *Q* with depth for the attenuation model. In this context, the shallow portion undergoes more pronounced attenuation, while the deeper segment displays weaker attenuation. Furthermore, in Fig. 5b, c, the vertical distribution of *S*-wave model points suggests minimal lateral variations in *S*-wave velocity models below 6 km. The velocity models appear more homogeneous, whereas the attenuation model still exhibits strong lateral variations. Despite the value of the slope, we also computed correlation coefficients (cc) to investigate whether the *Q* models exhibit a linear relationship with *V*, suggesting a correlation between attenuation structures and material characteristics. The resultant cc range from −0.1 to 0.61, with a decrease observed as a function of depth. Notably, it becomes evident that a perfect linear relationship does not exist, as the highest cc observed within the top layer is 0.61. These observations may suggest the presence of other significant factors, such as partial melts, fluid saturation in the pores of the medium, and/or elastic scattering caused by highly heterogeneous media, all of which may deteriorate the *Q*–*V* correlation^35,36^.

Laboratory investigations on olivine-rich rocks suggest that both velocity and attenuation exhibit strong temperature dependencies^37,38^. Regional^39^ and global studies^40^ have demonstrated a correlation between *V*_*S*_ and *Q*_*S*_, supported by robust correlation coefficients determined in statistical analyses. This implies that both attenuation and velocity anomalies primarily stem from thermal influences. However, regional studies^35^ have reported low correlation coefficients between *V* and *Q*, which contradict laboratory findings regarding the pronounced temperature dependence of *V* and *Q*. This disparity suggests that temperature alone cannot fully account for the variations in attenuation and velocity. Various physical mechanisms and artifacts associated with inversion may disrupt the expected correlations between *V* and *Q*. The presence of partial melts or fluids can notably impact velocity and attenuation on non-linear scales^37,41^. A modest proportion of partial melting notably influences shear modulus, resulting in a considerable decrease in shear wave velocity^36,42–44^, while exerting relatively minor effects on shear wave attenuations^42–44^. Consequently, the local occurrence of partial melts and/or fluids may undermine *Q*–*V* correlations. The anisotropy of seismic shear waves may also contribute to the inconsistency in the spatial variations of *Q* and *V* ^45^.

### High attenuation anomalies (low *Q*) beneath NTVZ

The crustal area displays 3-D *Q*_*P*_ and *Q*_*S*_ anomalies with amplitudes of ± 50% and strong lateral variations (Fig. 3). The notable consistency observed in the independently derived ${Q}_{P}^{-1}$ and ${Q}_{S}^{-1}$ models across all spatial dimensions provides strong evidence for the existence of significant structural features. The areas exhibiting high attenuation (low *Q*) anomalies for both *P* and *S* waves exhibit a strong correlation with the surface volcanoes along the northern Taiwan volcanic zone (NTVZ), as illustrated in Fig. 3a, b, e, f. Particularly, the most prominent low* Q*_*P*_ and *Q*_*S*_ anomalies, situated at depths of 1, 5, and 8 km, are found beneath the Tatun volcano group (TVG). When examining the cross-sections AA’ and BB’ in Fig. 4, it becomes evident that the low-*Q* anomaly extends vertically within a depth range of 1–10 km. Furthermore, depth-varying high-attenuation anomalies are also observed beneath other active offshore submarine volcanoes, including Keelungyu Island (KI) and Keelung volcano group (KVG) at depths ranging from 1 to 5 km, and Kueishan Island (KuI) at approximately 12 km depth (Figs. 3 and 4). These depth-dependent high-attenuation anomalies along the NTVZ are robust as confirmed through characteristic-model tests (Supplementary Figs. 6–9). While the strong attenuation features beneath the volcano regions could potentially be linked to hydrothermal events^46–48^ or magma reservoirs^49–51^, distinguishing the dominant mechanisms based solely on* Q*_*P*_ or *Q*_*S*_ data remains challenging. However, by leveraging additional independent constraints provided by *Q*_*P*_*/Q*_*S*_ and velocity (as detailed in "The ratio of to in the NTVZ"), we can effectively discern whether hydrothermal or magma activities are responsible for the low-*Q* anomalies in various regions.

Apart from the volcanic regions, the Yilan plain (YP), which extends westward from the Okinawa Trough^52^, displays relatively low-*Q* anomalies at depths ranging from 1 to 8 km (Fig. 3a, b). This observation suggests a potential association with sedimentary deposition in the region^52^. Nevertheless, the strength of the attenuation in these anomalies is weaker compared to the structures associated with volcanoes. We have successfully reconstructed the low*-Q* characteristic model of the Taipei and Yilan basins, which are the primary basins in northern Taiwan, demonstrating excellent recovery in all three dimensions (see Supplementary Figs. 12 and 13). This indicates that the dataset employed in this study has the capability to address the low-*Q* anomalies in the top 5-km layer within these two basins. The slightly low-*Q* anomalies observed in the Taipei and Yilan basins in our tomography model suggest that seismic attenuation is more sensitive to structures containing high-temperature materials than to soft and loose sediments.

### Low attenuation anomalies (high *Q*) and serpentinization

Our model also revealed a striking characteristic: low-attenuation (high *Q*) anomalies beneath the Hsuishan Range (HR, Fig. 1a) in the upper crust, specifically above a depth of 10 km (Fig. 3). Interestingly, previous travel-time tomographic studies have consistently reported high-velocity anomalies in the same region^12,31^. The coexistence of these high-*V* and high-*Q* anomalies suggests a potential correlation with the exhumation of metamorphic slate and argillite belts during the mountain-building process. Moreover, our attenuation models have revealed a notable high-*Q* feature in the southeastern offshore area of the Yilan plain, which is situated in the forearc region of the Ryukyu subduction zone (Figs. 3 and 4). This distinct low-attenuation anomaly spans a depth range from 5 km to approximately 10 km and exhibits *Q*_P_/*Q*_S_ ratios below 1, indicating the presence of partially saturated materials^53–55^. The geographical proximity of the forearc to the trench, characterized by heightened stress conditions^56^, raises the possibility of significant hydration and serpentinization processes^57^ playing a role in shaping the observed attenuation patterns^58^ in this region. The serpentinization of peridotites within the oceanic crust is frequently associated with the permeation of seawater through pre-existing faults and fractures^59,60^. The presence of active shallow faulting developments has been well-documented through geophysical surveys^61^ and seismicity studies^62^, which provide suitable pathways for seawater infiltration. Moreover, an alternative source for serpentinized peridotites in this context is the forearc mantle. A recent tomographic study^63^ indicated that the degree of serpentinization in the forearc wedge near northeastern Taiwan could potentially exceed 15%, corresponding to approximately 2% water content. Due to their relatively lower density, serpentinized materials have a propensity to migrate within the crust. The formation of serpentinite intrusions has been documented in various regions worldwide, prompting extensive research into the underlying processes and mechanisms driving their emergence^64,65^.

### The ratio of ${Q}_{P}$ to ${Q}_{S}$ in the NTVZ

Seismic attenuation is a multifaceted phenomenon arising from a complex interplay of absorption and scattering mechanisms, especially in volcanic regions. The primary goal of our study is not to precisely quantify the individual contributions of these mechanisms. Instead, we aim to identify potential factors contributing to seismic attenuation by analyzing various lines of evidence derived from diverse data sources and methodologies. Previous researches, specifically studies involving coda waves, has indicated that the dominant factor responsible for seismic energy dissipation in volcanic regions may be scattering effects^66,67^. Another possible contributor to the high attenuation anomalies observed in volcanic regions could be fluid-related effects^52^. We acknowledge that a challenge of our approach lies in the precise distinction between scattering effects and fluid-related contributions. However, by synthesizing the evidence and insights gathered from various studies, we can make significant strides in advancing our understanding of the underlying mechanisms at play.

An in-depth exploration of *Q*_*P*_*/Q*_*S*_ ratio behavior holds the potential to yield crucial insights into the origins of pronounced attenuation structures. This is due to the fact that *Q*_*P*_ and *Q*_*S*_ may exhibit non-monotonic variations with respect to the dominant mechanisms responsible for seismic energy dissipation. To ensure an unbiased assessment of the *Q*_*P*_*/Q*_*S*_ ratio, consistent damping and smoothing factors were applied during inversion, alongside comparable data coverage (Supplementary Figs. 14–16). Leveraging the robust and independent resolution of frequency-dependent *Q*_*P*_ and *Q*_*S*_ measurements, the *Q*_*P*_*/Q*_*S*_ emerges as a valuable tool for distinguishing between partially and fully fluid-saturated materials^47^. As previously discussed, we identified low-*Q* anomalies beneath the volcanic region in NTVZ, indicative of strong scattering attenuation, potential magma reservoirs, or hydrothermal activity. Notably, low *Q*_*P*_*/Q*_*S*_ ratios (*Q*_*P*_*/Q*_*S*_ ≈ 1) were exclusively observed beneath the TVG, while other volcanic regions within the NTVZ exhibited relatively high *Q*_*P*_*/Q*_*S*_ ratios (Fig. 3k–o). This discrepancy signifies a fundamental divergence in the underlying mechanisms governing energy dissipation. Hudson et al.^47^ employed *Q*_*P*_*/Q*_*S*_ ratios as a discriminating tool for materials' compressibility. Materials that are fully saturated or in a molten state are typically incompressible, resulting in *Q*_*P*_*/Q*_*S*_ ratios exceeding 1. Conversely, materials characterized by partial saturation tend to be more compressible, leading to *Q*_*P*_*/Q*_*S*_ ratios below 1. Therefore, the observed low-*Q* anomalies featuring low *Q*_*P*_*/Q*_*S*_ beneath the TVG region may be attributed to an active, shallow, and partially saturated hydrothermal system. While we acknowledge the possibility that low-*Q* anomalies could be attributed to strong scattering attenuation, as observed in other volcanic regions^68,69^, a recent waveform modeling study^46^ supports the hypothesis of active hydrothermal events in the TVG region favoring fluid-related contributions. Moreover, the existence of a magma reservoir beneath the TVG region remains plausible but may be situated at greater depths, as suggested by recent tomographic investigations^12^. These deeper magma reservoirs could serve as potential heat sources for the shallower hydrothermal system. Furthermore, this hydrothermal reservoir in the shallow crust is positioned in close proximity to a high fumarolic activity area within the TVG^39^, where volcanic degassing processes are notably robust. This underscores the potential for future phreatic eruptions in the region.

The relatively high *Q*_*P*_*/Q*_*S*_ ratios observed in other volcanoes within the NTVZ point toward the potential presence of melting materials, magma reservoirs. An interesting observation is that the ratios beneath KuI, at approximately 12 km depth, approach 2, which indicates that attenuation is predominantly governed by intrinsic absorption rather than the scattering mechanism. This suggests a likely connection to the presence of a magma reservoir^47^. The existence of this magma reservoir beneath Kul is substantiated by various observations, including *P*-wave delays, *S*-wave shadows^70^, detections of non-double-couple earthquakes^70^, and high-magnetic anomalies inferred from airborne magnetic surveys^71^. While the NTVZ is situated above the leading edge of the northward-subducting Philippine Sea Plate (PSP), it is paramount to note that the origins of the magma reservoirs discovered at shallower depths (< 15 km) beneath KI, KVG, and KuI may not be directly linked to subduction-induced partial melting materials. This is due to the absence of a clear conduit connecting our observed low-*Q* anomalies to a deeper melting source originating from the subducting PSP. Consequently, alternative mechanisms and processes must be considered to understand the generation and ascent of magma in these specific regions. Wang et al.^1^, in contrast to the subduction-induced partial melting hypothesis, proposed a distinct mechanism involving post-collisional delamination to account for the formation of the NTVZ. This hypothesis is rooted in the unique geochemical characteristics of NTVZ magmas, which show significant contributions from the asthenosphere and metasomatized subcontinental mantle. According to their model, the delamination process entailed the upwelling and subsequent melting of these mantle sources, ultimately leading to the formation of the NTVZ. This alternative perspective suggests that the NTVZ may have originated from processes independent of the subducting PSP, offering an alternative explanation for the observed geochemical signatures in the region.

## Conclusion

The study focuses on 3-D attenuation tomography to investigate magmatic and tectonic processes in northern Taiwan. We compared our attenuation model with the latest *P*-wave and *S*-wave velocity models in northern Taiwan and examined the spatial variations in *Q* and *V* across different depth layers. Our findings revealed that the spatial variations in ${Q}_{S}$ did not exhibit a consistent correlation with ${V}_{S}$ due to differences in attenuation sensitivity to temperature, fluid, and anisotropy effects. The correlation is good between ${Q}_{P}$ and ${V}_{P}$, particularly in the upper to mid crust. The low-attenuation (high *Q*) and high-velocity anomalies beneath the Hsuishan Range indicates potential correlation with the exhumation of metamorphic slate and argillite belts during mountain-building processes. Another high-*Q* feature in the forearc region of the Ryukyu subduction zone indicates partial saturation, likely due to serpentinization processes resulting from seawater infiltration or forearc mantle hydration. The findings shed light on complex geological features and provide insights into the crustal properties of northern Taiwan.

High-attenuation anomalies (low *Q*) beneath the Northern Taiwan Volcanic Zone (NTVZ) and offshore submarine volcanoes suggest the presence of magma reservoirs or hydrothermal activity at various depths. Notably, the TVG area exhibits uniquely low *Q*_*P*_*/Q*_*S*_ ratios, while other volcanic regions in the NTVZ display contrasting high ratios. This disparity suggests fundamental differences in energy dissipation mechanisms. The findings align with previous studies linking low *Q*_*P*_*/Q*_*S*_ ratios to partially saturated hydrothermal systems in the TVG region. The observation of high *Q*_*P*_*/Q*_*S*_ ratios in other volcanoes within the NTVZ implies the presence of melting materials or magma reservoirs. Specifically, a magma reservoir beneath Kul, located at approximately 12 km depth, is prominently depicted in the *Q* models and is substantiated by various geological observations. These findings contribute to our understanding of attenuation, and magma reservoir characteristics, ultimately enhancing our ability to assess volcanic hazards and better comprehend the behavior of volcanic systems.

## Methods

### The forward problem

We equated the log-ratio of *P*- or *S*-wave data type to the sum of three terms

2 $${d}_{ij}\left(f\right)\equiv {\text{ln}}\left[\frac{{A}_{ij}\left(f\right)}{{\widetilde{A}}_{ij}\left(f\right)}\right]={s}_{i}\left(f\right)-\pi f\Delta {t}_{ij}^{*}\left(f\right)+{r}_{j}\left(f\right),$$

where the data functional ${d}_{ij}\left(f\right)$ is the logarithm of the spectral ratio of the observed amplitude ${A}_{ij}(f)$ and the synthetic amplitude ${\widetilde{A}}_{ij}(f)$. The ${s}_{i}\left(f\right)$, ${r}_{j}\left(f\right)$, and $\Delta {t}_{ij}^{*}\left(f\right)$ represent the source, receiver, and path anomalies, respectively. The path anomaly is given by the integral in Eq. (3),

3 $$\Delta {t}_{ij}^{*}\left(f\right)={\int }_{{\mathcal{P}}_{ij}}{T}_{ij}\left({\text{x}}\right)\Delta q\left({\text{x}},f\right) d{\text{x}}.$$

which is a function of the 3-D coordinates along the ray path ${\mathcal{P}}_{ij}$. Here, ${T}_{ij}\left(\mathbf{x}\right)$ is the Fréchet kernel for the ray path, which is computed from the reference velocity model^27^ using ray theory. $\Delta q\left(\mathbf{x},f\right)$ is assumed to be a linear functional of the differential attenuation $\Delta q\left(\mathbf{x},f\right)\equiv {Q}^{-1}\left(\mathbf{x},f\right)-{\widetilde{Q}}^{-1}\left(z,f\right)$. The quantity ${Q}^{-1}\left({\text{x}},f\right)$ is the inverse of the *Q* model at the point **x** and frequency f, and ${\widetilde{Q}}^{-1}\left({\text{z}},f\right)$ is the initial 1-D model.

### Frequency- and depth-dependent attenuation model

Given the absence of high-resolution attenuation tomography in our study area, we derived the initial 1-D model for *Q* using attenuation-velocity scaling relationships. These relationships yielded ${Q}_{P}={Q}_{S}=75\times {V}_{S}$, where *V*_*S*_ represents the laterally averaged velocity from the tomography model published by Huang et al.^31^. We conducted separate inversions for *P* and *S* waves in 10 1-Hz frequency bands from 1 to 10 Hz. To characterize frequency-dependent effects in our synthetic simulations, we utilized a power law exponent *α* in Eq. (1) of approximately 0.5. The prior model distribution was constructed with a mean equivalent to that of the initial ${\widetilde{Q}}^{-1}$ model and an exponential covariance structure. Parameter variances were set to represent 50% relative uncertainties in ${Q}^{-1}$. The exponential scale factor was employed to effectively balance data fit and perturbation smoothness, following the methodology proposed by Lin and Jordon^21^. The source and receiver anomalies, denoted as ${s}_{i}$ and ${r}_{j}$ in Eq. (2), respectively, were assumed to follow independent distributions, both characterized by a prior mean of zero and prior variances without constraints on the inversion process. Data uncertainties were considered to be uncorrelated. To estimate the uncertainty of the data, we analyzed the scatter of residuals in epicentral-distance bins, as illustrated in Supplementary Fig. 4. The resulting models are shown in Fig. 2.

### Northern Taiwan attenuation tomography model

We extended our analysis beyond the one-dimensional model to investigate the three-dimensional model for the study area. The forward modeling process followed a similar approach to that of the 1-D problem. However, unlike the 1-D models solved in 10 frequency bands, we focused on spectral amplitudes at central frequency ${f}_{0}$. We corrected the spectral amplitudes to a common reference frequency (${f}_{0}=5$ Hz) using spatially-averaged exponents ${\alpha }_{P}=0.41$ and ${\alpha }_{S}=0.47$, as estimated from Eq. (1) (see Fig. 2). Assuming the estimated *α* values for the attenuation structure in the study area were homogeneous, we expressed $\Delta {t}_{ij}^{*}\left(f\right)$ in Eq. (3) by substituting Eq. (1)

4 $$\Delta {t}_{ij}^{*}\left(f\right)={\int }_{{\mathcal{P}}_{ij}}\left[{Q}^{-1}\left(\mathbf{x},f\right)-{\widetilde{Q}}^{-1}\left(\mathbf{x},f\right)\right]\times {T}_{ij}\left(\mathbf{x}\right)d\mathbf{x}={\int }_{{\mathcal{P}}_{ij}}{\left(\frac{f}{{f}_{0}}\right)}^{-\alpha }\left[{Q}_{0}^{-1}(\mathbf{x})-{\widetilde{Q}}_{0}^{-1}(\mathbf{x})\right] {T}_{ij}(\mathbf{x})d\mathbf{x}.$$

By utilizing amplitude measurements across all frequency bands, we can perform a joint frequency bands inversion for 3-D* Q* perturbations at the central frequency ${f}_{0}$, represented as $\Delta {q}_{0}\left(\mathbf{x}\right)\equiv {Q}_{0}^{-1}(\mathbf{x})-{\widetilde{Q}}_{0}^{-1}(\mathbf{x})$ in Eq. (4). A major advantage of this multi-frequency approach is the ability to compute frequency-dependent source and station residuals with *Q* structures simultaneously (Eq. 2), which effectively reduces the trade-off issue between the source model and *Q* structures^14^. The frequency-dependent receiver statics^21^ can be described by a linear function ${r}_{j}\left(f\right)={r}_{j0}-\pi {\kappa }_{j}\left(f-{f}_{0}\right)$, resulting in unknown model parameters of station response (10-frequency bands × *j* stations) being reduced to 2 × *j* (${r}_{j0}$ and ${\kappa }_{j})$. This simplification of station residuals helps to reduce computation time. To perform the joint inversion of multi-frequency data, we can re-write Eq. (2) as

5 $${d}_{ij}\left(f\right)\equiv {\text{ln}}\left[\frac{{A}_{ij}\left(f\right)}{{\widetilde{A}}_{ij}\left(f\right)}\right]={s}_{i}\left(f\right)-\pi f{\left(\frac{f}{{f}_{0}}\right)}^{-\alpha }{\int }_{{\mathcal{P}}_{ij}}^{ }\left[{Q}_{0}^{-1}\left(\mathbf{x}\right)-{\widetilde{Q}}_{0}^{-1}\left(\mathbf{x}\right)\right]{T}_{ij}\left(\mathbf{x}\right)d\mathbf{x}+{r}_{j0}-\pi {\kappa }_{j}\left(f-{f}_{0}\right).$$

We formulate the data equation as a linear system, $\mathbf{G}\mathbf{m}=\mathbf{d}$, where the model vector **m** includes the differential attenuation parameters $\Delta {q}_{0}\left(\mathbf{x}\right)$, source statics ${s}_{i}(f)$, and receiver statics ${r}_{j0}$ and ${\kappa }_{j}$. We determine the power law rate *α* from 1-D *Q* models in frequency bands from 1 to 10 Hz (Eq. 1) and use it to estimate the 3-D *Q* perturbations at the central frequency (${f}_{0}=5$ Hz) (Eq. 5). The initial attenuation model is from our 1-D model at 5 Hz, ${\widetilde{Q}}_{0}^{-1}\left(\mathbf{x}\right)={Q}^{-1}\left({\text{z}},5\right)$ (green solid curves in Fig. 2a, b).

In 3-D tomography calculation, we also conducted separate inversions for *P* and *S* waves. The prior means were the updated 5-Hz models of Fig. 2 and the prior covariances were exponential with 50% relative uncertainties and specified lateral and radial smoothing lengths. The station statics ${\kappa }_{j}$ represent the residual signal between the observed and predicted amplitude at each station, after accounting for the attenuation model. To ensure that the *Q* perturbations capture as much of the true signal as possible, the prior variance of ${\kappa }_{j}$ was purposely chosen to be small, which means that the station statics were strongly constrained in the inversion. The prior information was built into the log-ratio data, resulting in source statics and attenuation differentials judged to have a zero prior mean for all parameters. The model vector **m** concatenated the differential attenuation parameters, source statics, and receiver statics, with a prior mean $\overline{\mathbf{m} }$ and prior covariance matrix ${\mathbf{C}}_{m}$. The observed data vector was assumed to sample a Gaussian process with a mean $\overline{\mathbf{d} }=\langle \mathbf{d}\rangle$ and a covariance matrix ${\mathbf{C}}_{d}=\langle (\mathbf{d}-\overline{\mathbf{d} }){(\mathbf{d}-\overline{\mathbf{d} })}^{{\text{T}}}\rangle$, with a linear system $\mathbf{G}\mathbf{m}=\mathbf{d}$. The resulting models are shown in Figs. 3 and 4.

### Supplementary Information

Supplementary Information.

## Data Availability

All the waveform data are available on request by Formosa Array (https://fmarray.earth.sinica.edu.tw/). Researchers can register for an account to apply for the data.
