# Supplementary material for: Unveiling attenuation structures in the northern Taiwan volcanic zone
Source: Sci Rep. 2024 Feb 27;14:4716. doi: 10.1038/s41598-024-55397-8 (PMC10899250; doi:10.1038/s41598-024-55397-8)
Supplement: Supplementary file 1 — Supplementary Information. [file 41598_2024_55397_MOESM1_ESM.docx]

**Supplementary Information for**

**Unveiling Attenuation Structures in the Northern Taiwan Volcanic Zone**

Lin, Y.-P.^1^, Ko, J. Y.-T.^2*^, Huang, B.-S.^3^, Lin, C.H.^3^, M.-H. Shih^4^

1. Department of Geosciences, National Taiwan University, Taipei, Taiwan
2. Institute of Oceanography, National Taiwan University, Taipei, Taiwan
3. Institute of Earth Sciences, Academia Sinica, Taipei, Taiwan
4. National Center for Research on Earthquake Engineering, Taipei, Taiwan

* Corresponding Author: Ko, J. Y.-T. (justinko@ntu.edu.tw)

**List of Supplementary Figures and Table:**

**Supplementary Fig. 1** P-wave and S-wave waveforms and corresponding spectra

**Supplementary Fig. 2** Frequency-dependent 1-D attenuation models derived from P waveforms obtained using four distinct time window lengths.

**Supplementary Fig. 3** Frequency-dependent 1-D attenuation models derived from S waveforms obtained using four distinct time window lengths.

**Supplementary Fig. 4** P-wave and S-wave amplitude residuals relative to the initial and the final model.

**Supplementary Fig. 5** Checkerboard recovery test for *P*-wave and *S*-wave attenuation (20 km × 20 km × 10 km).

**Supplementary Fig. 6** Depth slices of the trial model and model recovery for low-*Q* anomaly in Tatun volcano

**Supplementary Fig. 7** Cross sections of the trial model and model recovery for low-*Q* anomaly in Tatun volcano

**Supplementary Fig. 8** Depth slices of the trial model and model recovery for low-*Q* anomaly beneath Kueishan Island (KuI)

**Supplementary Fig. 9** Cross sections of the trial model and model recovery for low-*Q* anomaly beneath Kueishan Island (KuI)

**Supplementary Fig. 10** Depth slices of the trial model and model recovery for low-*Q* anomalies beneath Keelungyu Island (KI) and Keelung volcano group (KVG)

**Supplementary Fig. 11** Cross sections of the trial model and model recovery for low-*Q* anomalies beneath Keelungyu Island (KI) and Keelung volcano group (KVG)

**Supplementary Fig. 12** Depth slices of the trial model and model recovery for low-*Q* anomalies in Taipei and Yilan basins

**Supplementary Fig. 13** Cross sections of the trial model and model recovery for low-*Q* anomalies in Taipei and Yilan basins

**Supplementary Fig. 14** Depth slices of the trial model and model recovery for low- and high-*Q_P_* anomalies in northern Taiwan

**Supplementary Fig. 15** Depth slices of the trial model and model recovery for low- and high-*Q_S_* anomalies in northern Taiwan

**Supplementary Fig. 16** Depth slices of the trial model and model recovery for *Q_P_*/*Q_S_* anomalies in northern Taiwan

**Supplementary Fig. 17** *Q_S_* vs. *Q_P_*

**Supplementary Table 1** Parameters used in *Q_P_*/*Q_S_* recovery test in northern Taiwan


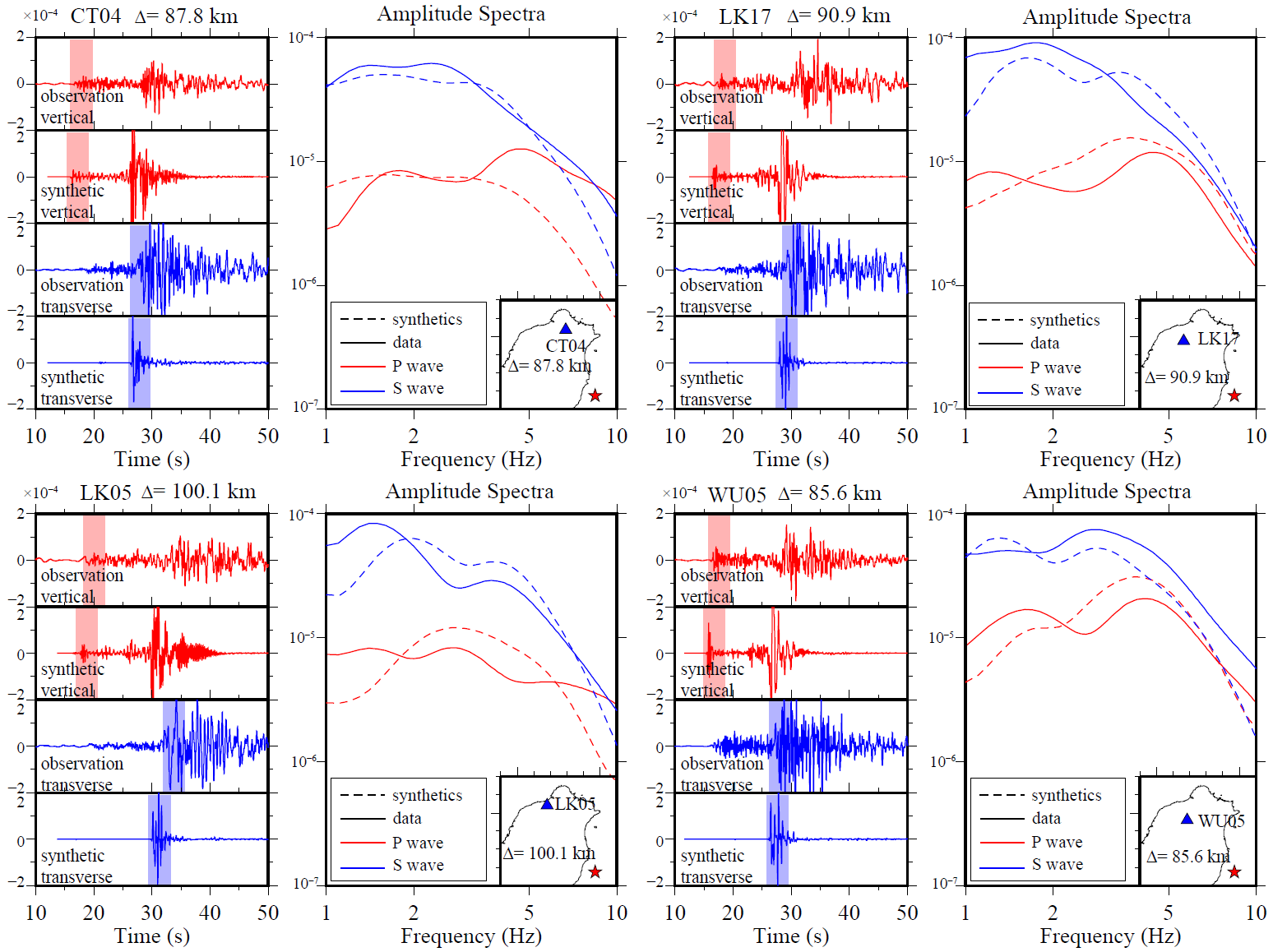


**Supplementary Fig. 1** Four synthetic waveforms (left panels) and corresponding spectra (dashed lines in right panels) are juxtaposed with observed waveforms (left panels) and spectra (solid lines in right panels) for *P* waves (red lines) and *S* waves (blue lines). The recorded spectra originate from four seismic stations, and their respective earthquake epicenter locations are marked on the inset maps. Shaded areas denote the time windows (3.7 sec) used for amplitude measurements of *P* and *S* waves. All spectra are derived from the time-integrated wavelet transform.


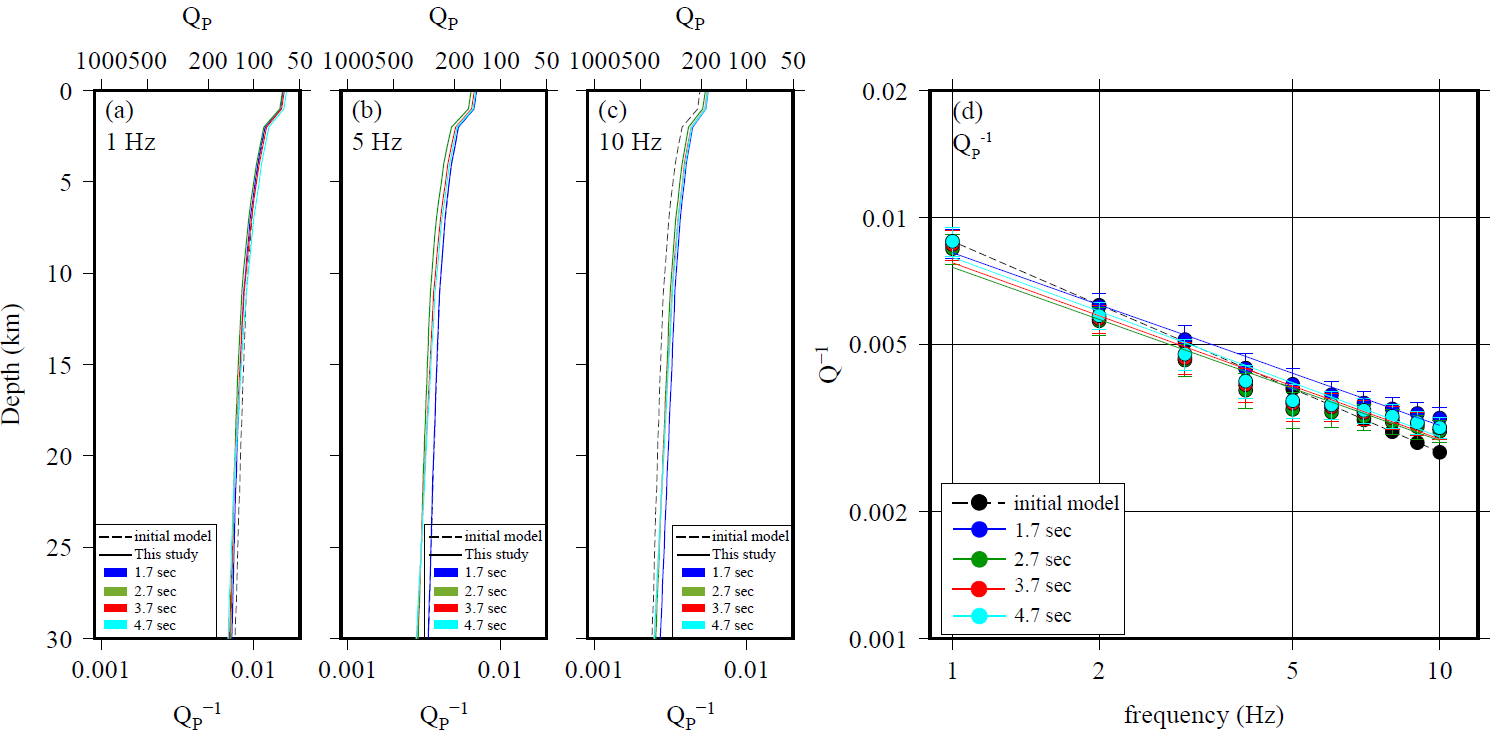


**Supplementary Fig. 2** Frequency-dependent 1-D attenuation models derived from *P* waveforms obtained using four distinct time window lengths. Panels (a)-(c) display depth-dependent *Q_P_* models at 1 Hz, 5 Hz, and 10 Hz, respectively. The colored solid lines represent *Q* models based on *P* waveforms measured within the four different time-window lengths. Panel (d) illustrates the frequency-dependent *Q* models derived from *P* waveforms with these varying time-window lengths. Despite examining the models, no significant differences are discernible; they mostly overlap, except for a slight offset observed with the 1.7 s length measurements. This shortest time window may not encompass the entire direct *P*-wave energy. For this attenuation model study, waveforms were measured within a 3.7-second time window.


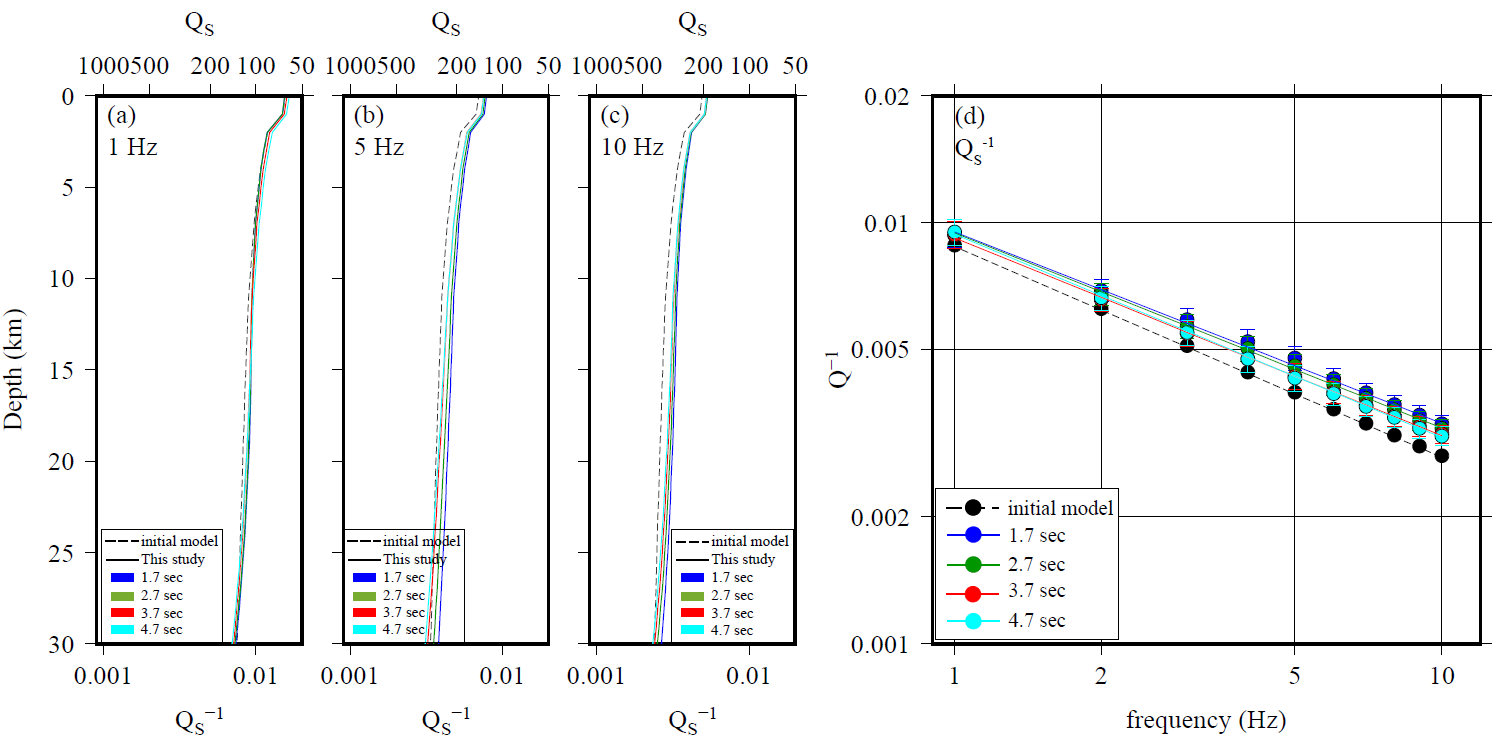


**Supplementary Fig. 3** Frequency-dependent 1-D attenuation models derived from *S* waveforms obtained using four distinct time window lengths. Panels (a)-(c) display depth-dependent *Q_S_* models at 1 Hz, 5 Hz, and 10 Hz, respectively. The colored solid lines represent *Q* models based on *S* waveforms measured within the four different time-window lengths. Panel (d) illustrates the frequency-dependent *Q* models derived from *S* waveforms with these varying time-window lengths. Despite examining the models, no significant differences are discernible; they mostly overlap, except for a slight offset observed with the 1.7 s length measurements. This shortest time window may not encompass the entire direct *S*-wave energy. For this attenuation model study, waveforms were measured within a 3.7-second time window.


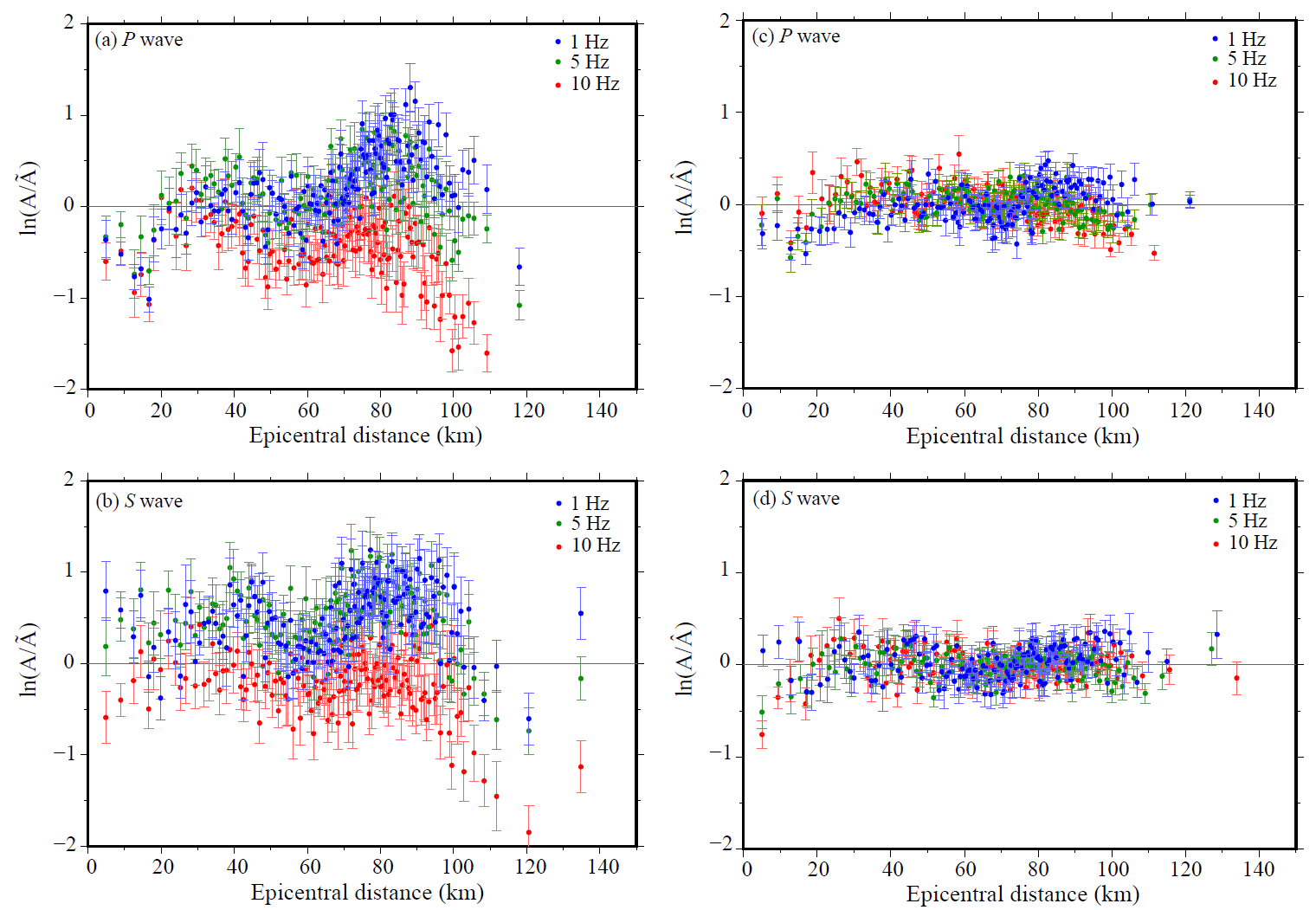


**Supplementary Fig. 4** Means (dots) and standard errors of the mean (vertical bars) of the data versus epicentral distance, obtained for contiguous 25-sample groupings at frequencies of 1 Hz (blue), 5 Hz (green), and 10 Hz (red) before and after the inversion. (a) *P*-wave and (b) *S*-wave residuals relative to the starting model. (c) *P*-wave and (d) *S*-wave residuals relative to the final 3-D model.


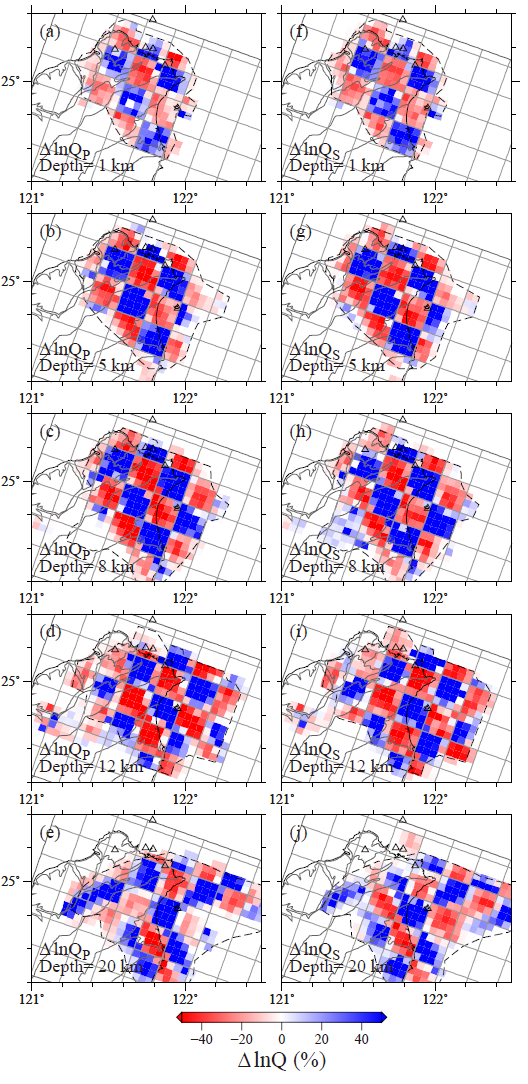


**Supplementary Fig. 5** Checkerboard recovery test for *P*-wave **(a)-(e)** and *S*-wave **(f)-(j)** attenuation. Checkerboard cell dimensions are 20 km × 20 km × 10 km (grey grids). Input perturbations are $\Delta\ln Q=\pm50\%$. Black dashed contours enclose the region where the inverted model recovers the perturbation with an amplitude greater than ±10%.


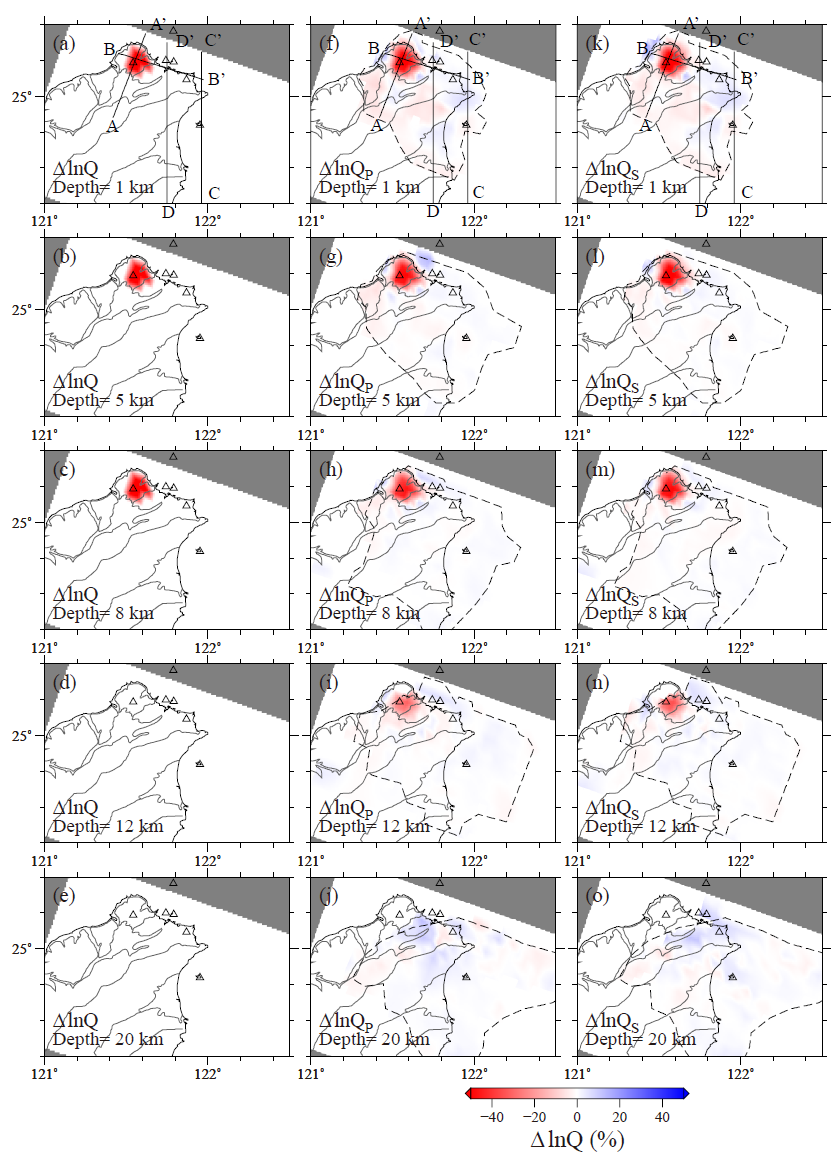


**Supplementary Fig. 6** **(a)-(e)** Depth slices of the trial model for strong attenuation (low *Q*) in Tatun volcano in northern Taiwan, which has constant, $-50\%$ perturbations to 10 km depth and no perturbations below this depth. **(f)-(o)**  $Q_{P}$ and $Q_{S}$ model recovered by inversion of the synthetic dataset calculated from the trial model of (a)-(e). Black dashed enclose the high resolution region from the 20 km × 20 km checkerboard test (Supplementary Fig. 5).


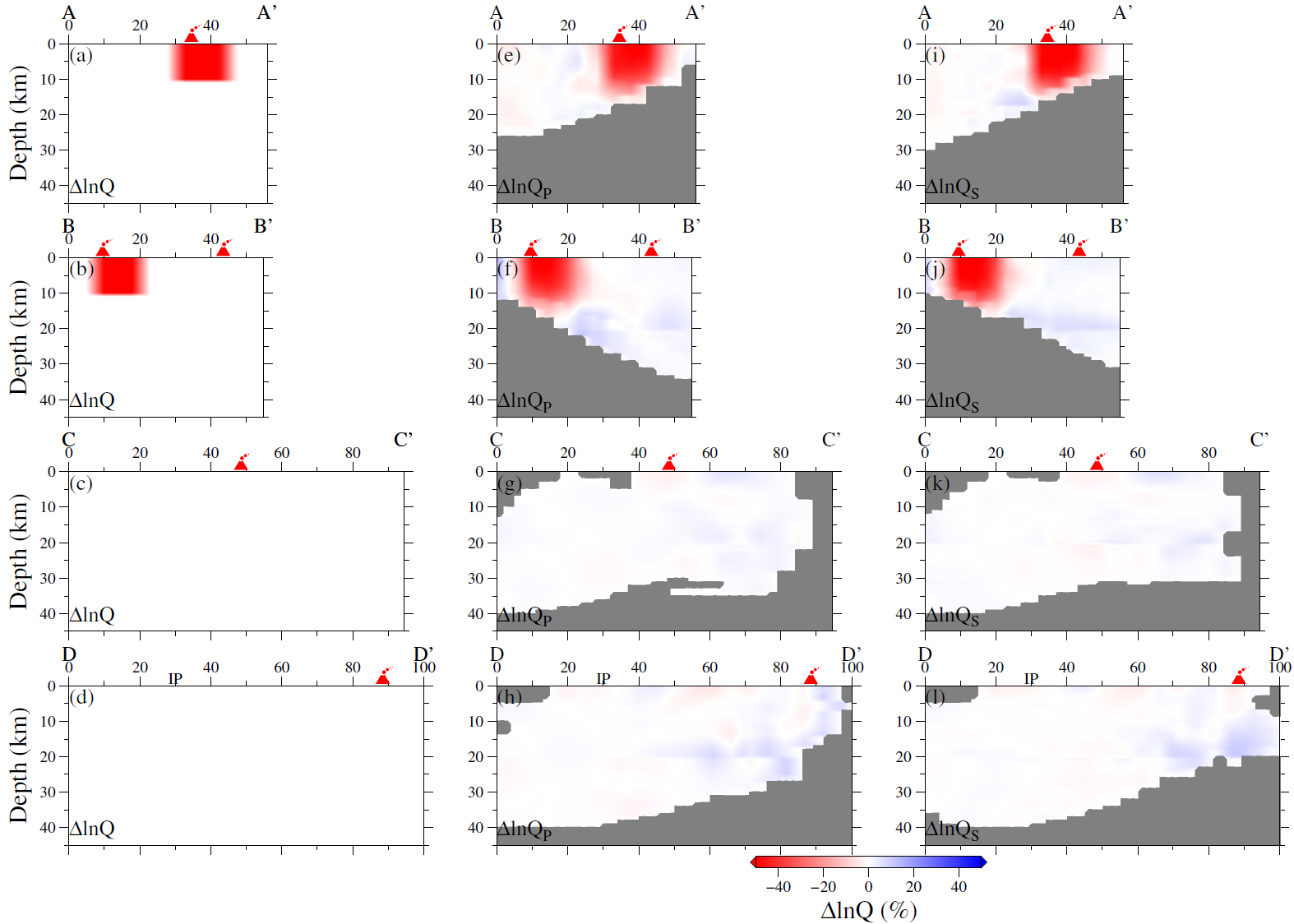


**Supplementary Fig. 7** **(a)-(d)** Cross sections of the trial model along lines A-A' to D-D' located on Supplementary Fig. 6a. for strong attenuation (low *Q*) in Tatun volcano in northern Taiwan, which has constant, $-50\%$ perturbations to 10 km depth and no perturbations below this depth. **(e)-(l)** $Q_{P}$ and $Q_{S}$ model recovered by inversion of the synthetic dataset calculated from the trial model of (a)-(d).


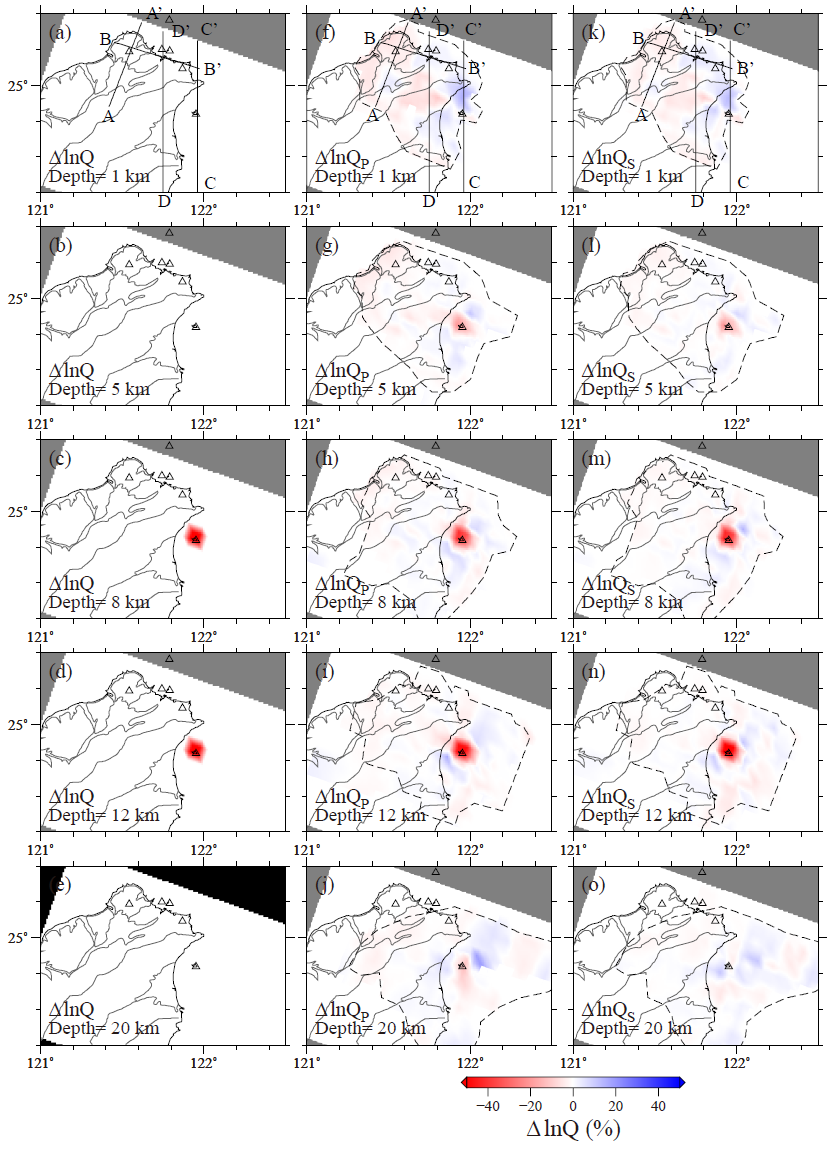


**Supplementary Fig. 8** **(a)-(e)** Depth slices of the trial model for strong attenuation (low *Q*) in Kueishan Island (KuI) in northern Taiwan, which has constant, $-50\%$ perturbations from 8 to 15 km depth and no perturbations above and below this range of depth. **(f)-(o)**  $Q_{P}$ and $Q_{S}$ model recovered by inversion of the synthetic dataset calculated from the trial model of (a)-(e). Black dashed enclose the high resolution region from the 20 km × 20 km checkerboard test (Supplementary Fig. 5).


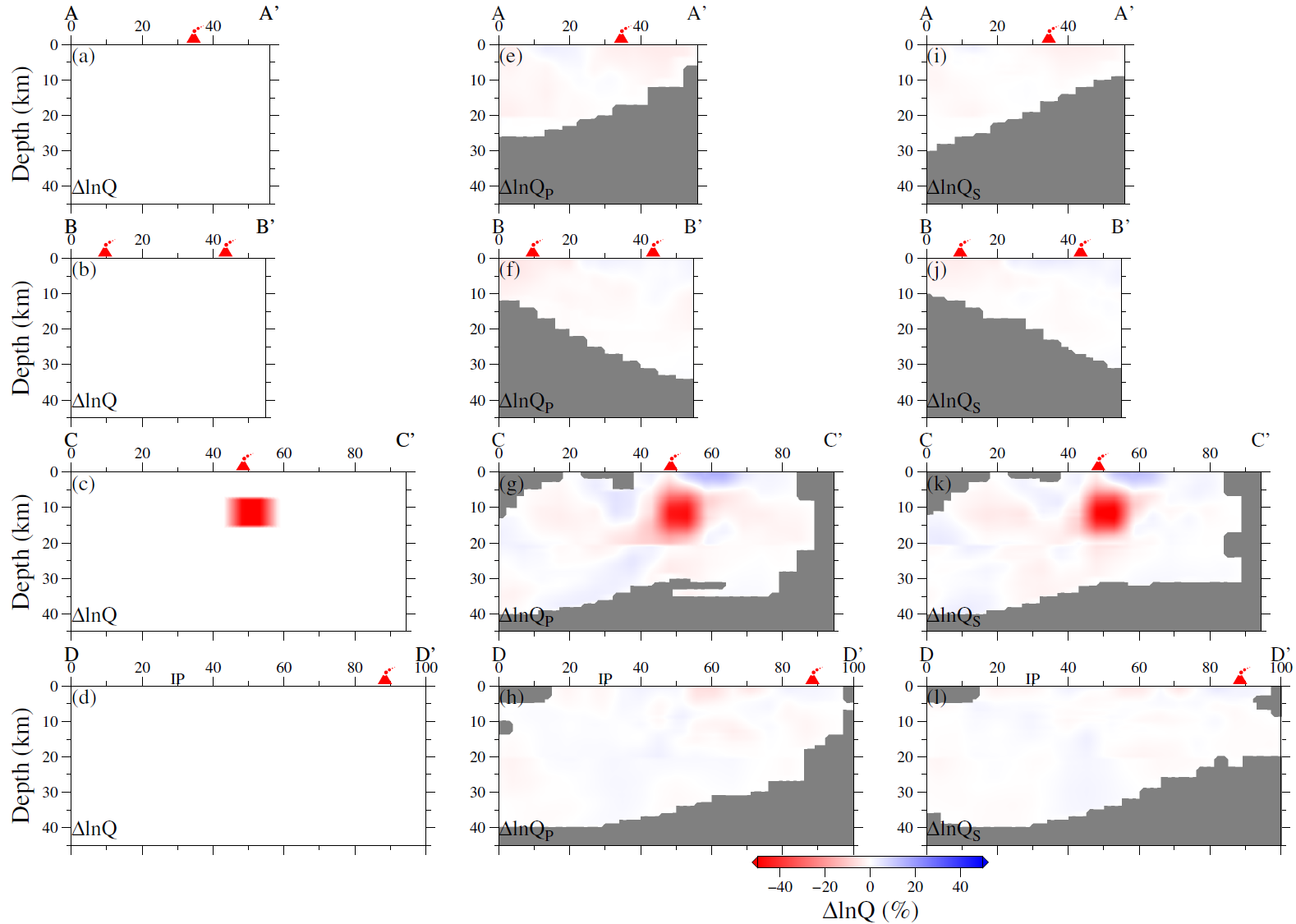


**Supplementary Fig. 9** **(a)-(d)** Cross sections of the trial model along lines A-A' to D-D' located on Supplementary Fig. 8a. for strong attenuation (low *Q*) in Kueishan Island (KuI) Island in northern Taiwan, which has constant, $-50\%$ perturbations from 8 to 15 km depth and no perturbations above and below this range of depth. **(e)-(l)** $Q_{P}$ and $Q_{S}$ model recovered by inversion of the synthetic dataset calculated from the trial model of (a)-(d).


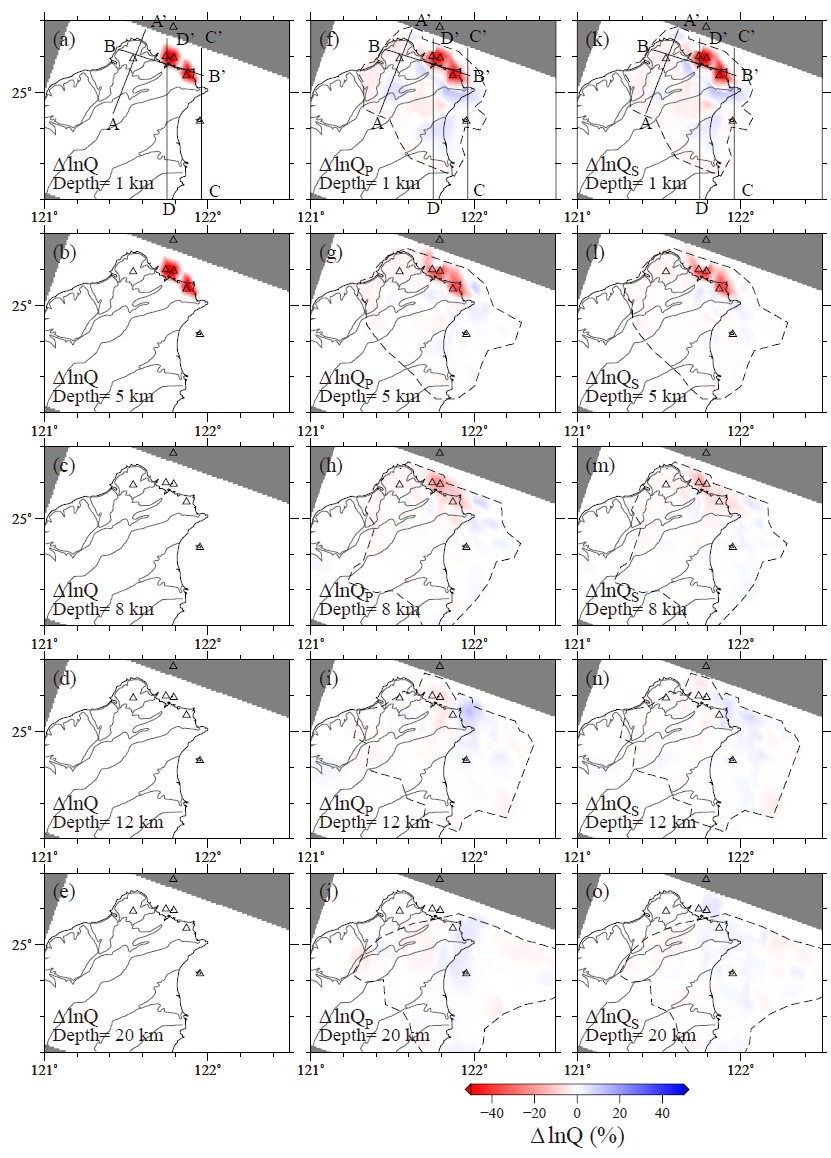


**Supplementary Fig. 10** **(a)-(e)** Depth slices of the trial model for strong attenuation (low *Q*) in Keelungyu Island (KI) and Keelung volcano group (KVG), which has constant, $-50\%$ perturbations to 5 km depth and no perturbations below this depth. **(f)-(o)**  $Q_{P}$ and $Q_{S}$ model recovered by inversion of the synthetic dataset calculated from the trial model of (a)-(e). Black dashed enclose the high resolution region from the 20 km × 20 km checkerboard test (Supplementary Fig. 5).


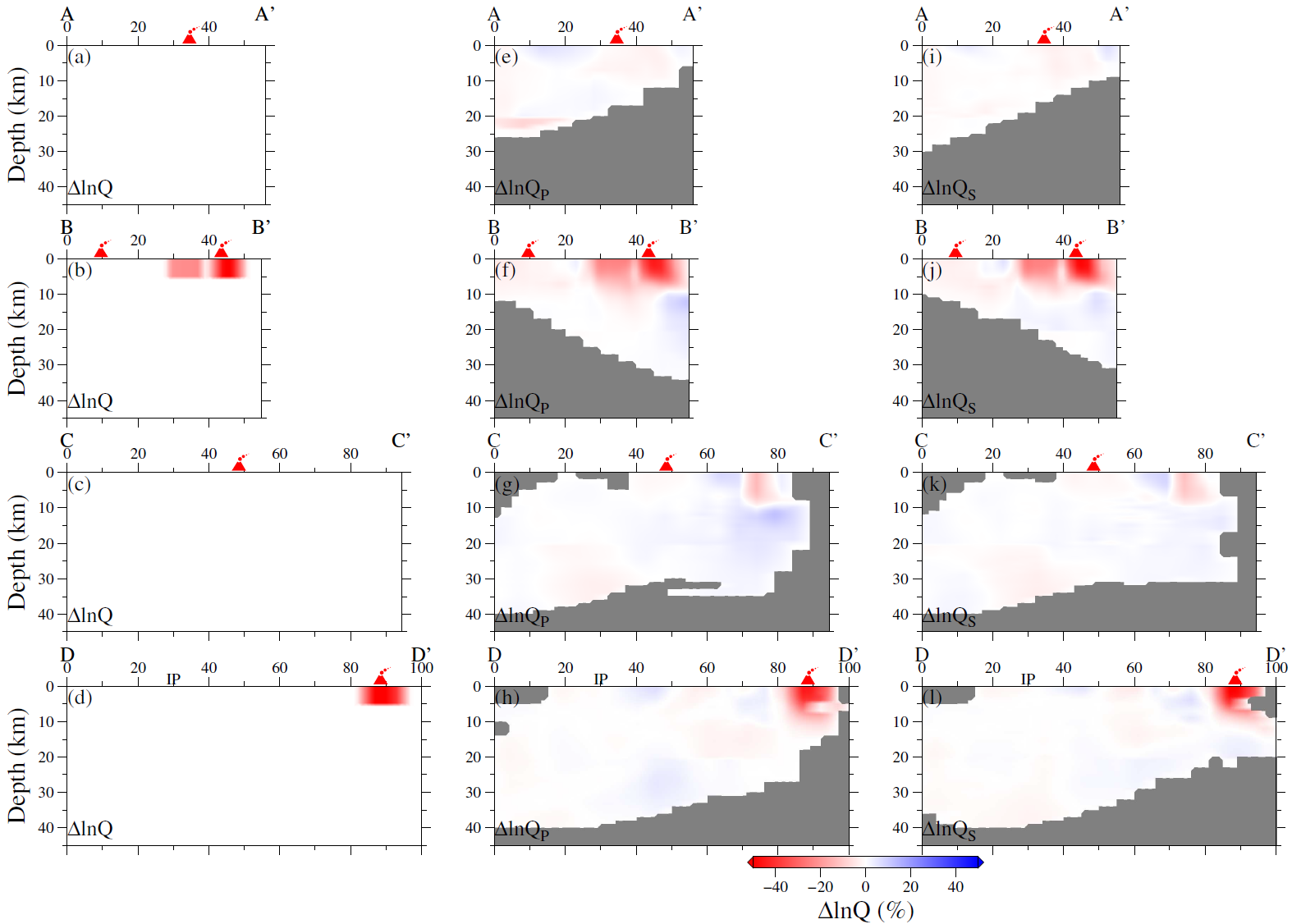


**Supplementary Fig. 11** **(a)-(d)** Cross sections of the trial model along lines A-A' to D-D' located on Supplementary Fig. 10a. for strong attenuation (low *Q*) in Keelungyu Island (KI) and Keelung volcano group (KVG) in northern Taiwan, which has constant, $-50\%$ perturbations to 5 km depth and no perturbations below this depth. **(e)-(l)** $Q_{P}$ and $Q_{S}$ model recovered by inversion of the synthetic dataset calculated from the trial model of (a)-(d).


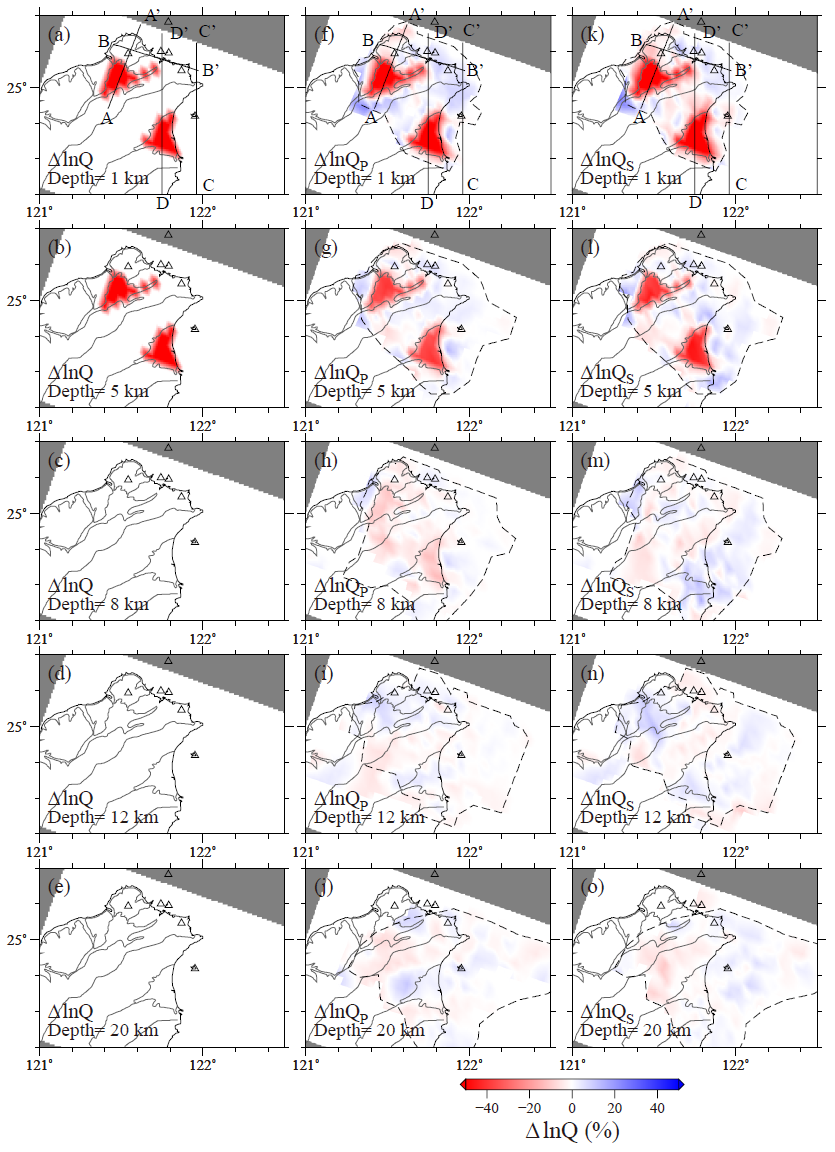


**Supplementary Fig. 12** **(a)-(e)** Depth slices of the trial model for strong attenuation (low *Q*) in Taipei and Yilan basins in northern Taiwan, which has constant, $-50\%$ perturbations to 5 km depth and no perturbations below this depth. **(f)-(o)**  $Q_{P}$ and $Q_{S}$ model recovered by inversion of the synthetic dataset calculated from the trial model of (a)-(d). Black dashed enclose the high resolution region from the 20 km × 20 km checkerboard test (Supplementary Fig. 5).


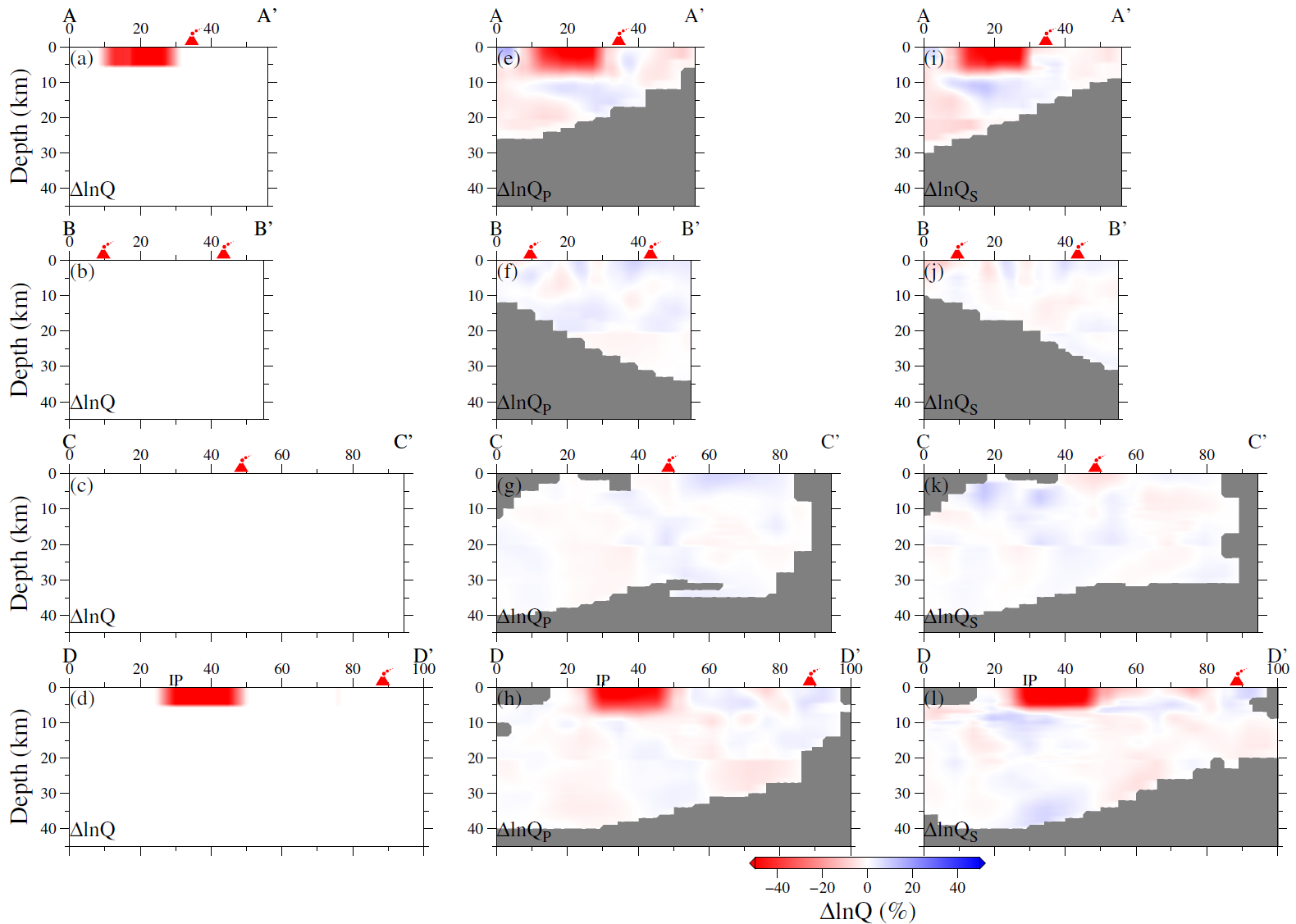


**Supplementary Fig. 13** **(a)-(d)** Cross sections of the trial model along lines A-A' to D-D' located on Supplementary Fig. 12a. for strong attenuation (low *Q*) in Taipei and Yilan basins in northern Taiwan, which has constant, $-50\%$ perturbations to 5 km depth and no perturbations below this depth. **(e)-(l)** $Q_{P}$ and $Q_{S}$ model recovered by inversion of the synthetic dataset calculated from the trial model of (a)-(d).

***Q_P_*/*Q_S_* recovery test**

The *Q_P_*/*Q_S_* ratio is a crucial parameter for determining structural characteristics. However, direct determination of this ratio through inversion is not feasible. Instead, we employ the same data, model parameterization, and regularization to independently solve for *Q_P_* and *Q_S_* models, from which we obtain the *Q_P_*/*Q_S_* map. This test aims to validate the recovery of the *Q_P_*/*Q_S_* map through separate inversion of *P*-wave and *S*-wave attenuation models. Supplementary Table 1 provides detailed information about the locations and intensities of anomalies in the input models, which are adopted from the key observations in our inverted attenuation models. The depth slices of both input and recovered *Q_P_*, *Q_S_*, and *Q_P_*/*Q_S_* are presented in Supplementary Figures 14-16, respectively. This test demonstrates the robust recovery of *Q_P_*/*Q_S_* maps despite the inherent challenges posed by differences in ray paths and travel times between *P*- and *S*-wave inversions. The results indicate that, even with these discrepancies, we can successfully recover the *Q_P_*/*Q_S_* map through independent inversions of *P*-wave and *S*-wave attenuation models. Consequently, the *Q_P_*/*Q_S_* observations in this study are reliable.

**Supplementary Table 1**

|  | Tatun volcano group  (TVG) | Kueishan Island  (KuI) | Offshore of  the Yilan Plain (YP) |
| --- | --- | --- | --- |
| Perturbation depth (km) | 0-10 | 8-15 | 5-15 |
| *Q_P_* | 160 | 240 | 400 |
| *Q_S_* | 160 | 120 | 800 |
| *Q_P_*/*Q_S_* | 1 | 2 | 0.5 |


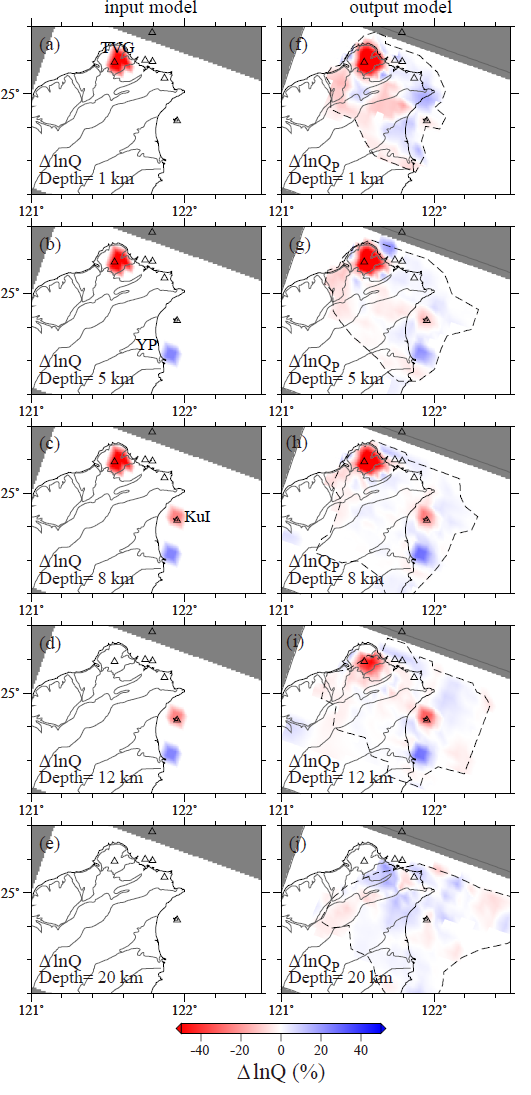


**Supplementary Fig. 14** (a)-(e) Depth slices of the trial model for strong *P*-wave attenuation (low *Q*) in Tatun volcano group (TVG) and Kueishan Island (KuI), and weak *P*-wave attenuation (high *Q*) in the region offshore of the Yilan plain (YP). The detailed input values of the attenuation anomalies are show in the Supplementary Table 1. (f)-(j) *Q_P_* model recovered by inversion of the synthetic dataset calculated from the trial model of (a)-(e). Black dashed enclose the high resolution region from the 20 km × 20 km checkerboard test (Supplementary Fig. 5). The reference *Q* value is 320.


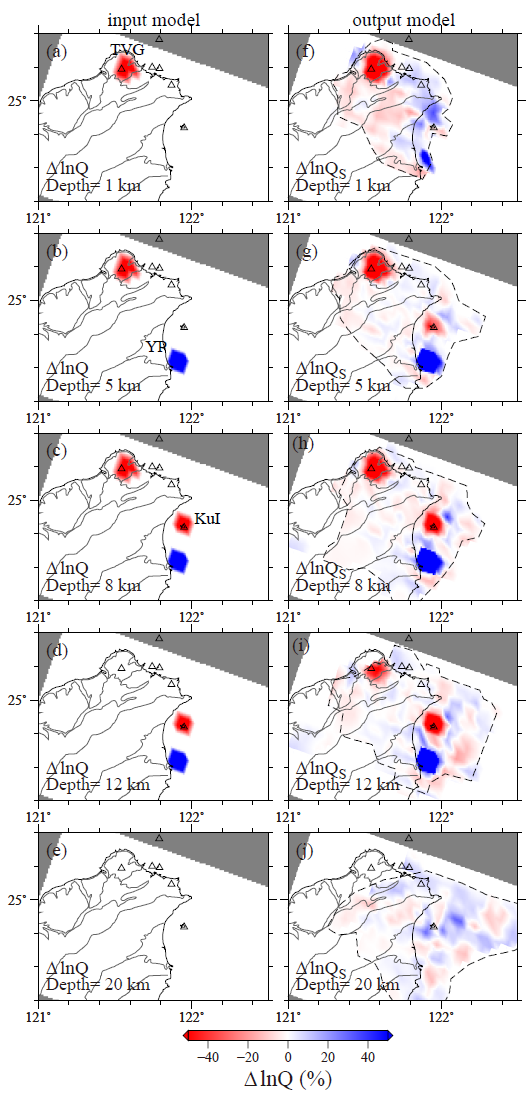


**Supplementary Fig. 15** **(a)-(e)** Depth slices of the trial model for strong *S*-wave attenuation (low *Q*) in Tatun volcano group (TVG) and Kueishan Island (KuI), and weak *S*-wave attenuation (high *Q*) in the region offshore of the Yilan plain (YP). The detailed input values of the attenuation anomalies are show in the Supplementary Table 1. **(f)-(j)**  $Q_{S}$ model recovered by inversion of the synthetic dataset calculated from the trial model of (a)-(e). Black dashed enclose the high resolution region from the 20 km × 20 km checkerboard test (Supplementary Fig. 5). The reference *Q* value is 320.


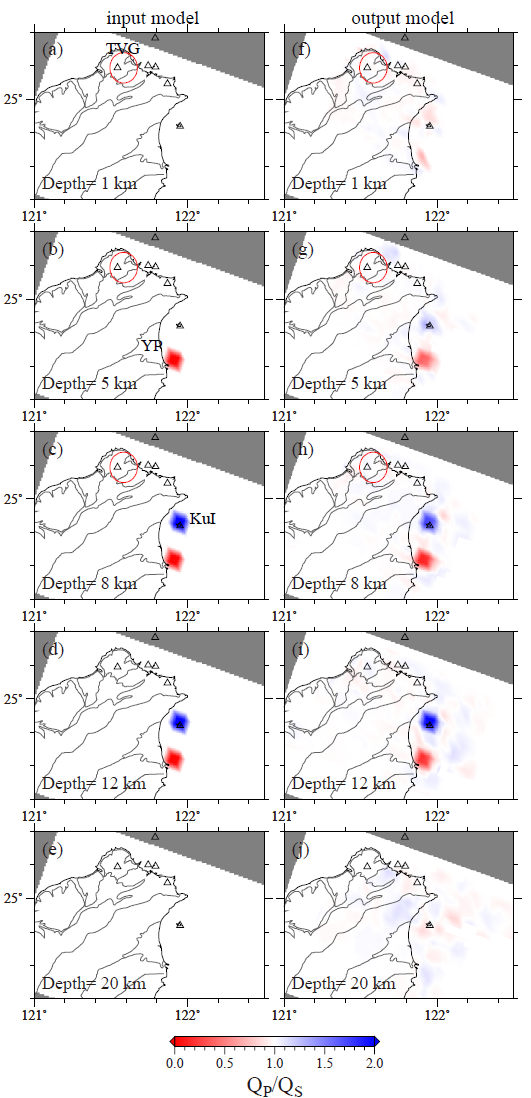


**Supplementary Fig. 16** **(a)-(e)** Depth slices of the trial model for *Q_P_*/*Q_S_*. The ratio of the trial model is 1 in the top 10-km thickness layer beneath Tatun volcano group (TVG), where white represents this specific ratio. A red circle is utilized to pinpoint the location of the anamoly under the TVG. The ratio is 2 under the Kueishan Island (KuI), and 0.5 in the region offshore of the Yilan plain (YP). The detailed parameters of the attenuation anomalies are show in the Supplementary Table 1. **(f)-(j)** *Q_P_*/*Q_S_* maps derived from independent inversions of *Q_P_* and *Q_S_* models (see panels (f)-(j) in Supplementary Figures 14 and 15). The consistent ratios of these anomalies between input and ouput maps underscore the robustness of the *Q_P_*/*Q_S_* observations.


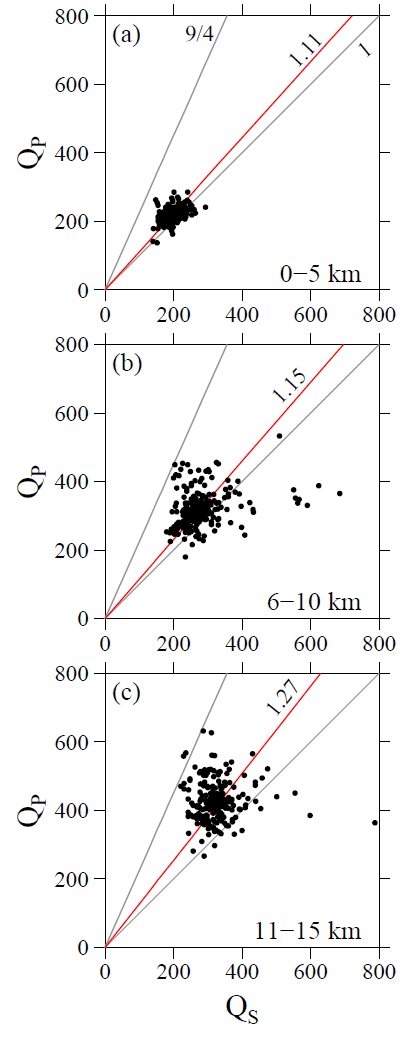


**Supplementary Fig. 17** *Q_S_* vs. *Q_P_* for layer averages of geographic cells in the high- resolution region. Slopes of best-fitting lines (*Q_P_*/*Q_S_*) are labeled. The linear relationship between *Q_P_* and *Q_S_* is not strongly pronounced, yet the majority of *Q* model points are concentrated, indicating that both *Q_P_* and *Q_S_* values fall within a similar *Q* range. Given the evident lateral variations in the *Q_P_*/*Q_S_* model, it can be anticipated that the distribution of *Q_S_* versus *Q_P_* will not be perfectly linear. However, upon reviewing Figures 3 and 4, the primary regions of interest that we discuss, such as TVG, NTVZ, Hsuishan Range, and the offshore area of the Yilan plain, The *Q_P_* and *Q_S_* models show the similar lateral variations. In northern Taiwan, average *Q_P_*/*Q_S_* values consistently exceed 1 from the shallow to deep layers. This observation also implies that elastic scattering is not the primary mechanism governing the attenuation model in northern Taiwan.
